# Continuum microhaemodynamics modelling using inverse rheology

**DOI:** 10.1007/s10237-021-01537-2

**Published:** 2021-12-14

**Authors:** Joseph van Batenburg-Sherwood, Stavroula Balabani

**Affiliations:** 1 Department of Bioengineering, Imperial College London, London, UK; 2 Department of Mechanical Engineering, University College London, London, UK

**Keywords:** Blood flow, Haemorheology, Red blood cells, Continuum modelling, Data assimilation, Inverse rheology, Particle image Velocimetry, Computational fluid dynamics, Microfluidics

## Abstract

Modelling blood flow in microvascular networks is challenging due to the complex nature of haemorheology. Zero- and one-dimensional approaches cannot reproduce local haemodynamics, and models that consider individual red blood cells (RBCs) are prohibitively computationally expensive. Continuum approaches could provide an efficient solution, but dependence on a large parameter space and scarcity of experimental data for validation has limited their application. We describe a method to assimilate experimental RBC velocity and concentration data into a continuum numerical modelling framework. Imaging data of RBCs were acquired in a sequentially bifurcating microchannel for various flow conditions. RBC concentration distributions were evaluated and mapped into computational fluid dynamics simulations with rheology prescribed by the Quemada model. Predicted velocities were compared to particle image velocimetry data. A subset of cases was used for parameter optimisation, and the resulting model was applied to a wider data set to evaluate model efficacy. The pre-optimised model reduced errors in predicted velocity by 60% compared to assuming a Newtonian fluid, and optimisation further reduced errors by 40%. Asymmetry of RBC velocity and concentration profiles was demonstrated to play a critical role. Excluding asymmetry in the RBC concentration doubled the error, but excluding spatial distributions of shear rate had little effect. This study demonstrates that a continuum model with optimised rheological parameters can reproduce measured velocity if RBC concentration distributions are known *a priori*. Developing this approach for RBC transport with more network configurations has the potential to provide an efficient approach for modelling network-scale haemodynamics.

**Supplementary Information:**

The online version contains supplementary material available at 10.1007/s10237-021-01537-2.

## Introduction

Cardiovascular diseases are the foremost cause of death globally, and central to many of these conditions are altered blood flow dynamics. Understanding haemodynamics and being able to accurately model it using numerical methods is therefore of importance for both research and clinical applications.

Blood rheology is determined predominantly by the red blood cells (RBCs), which are suspended in the plasma at a volume concentration (haematocrit) that varies throughout the vasculature, with an average of 45% in large vessels. The specialised, anuclear RBCs are highly deformable, allowing them to fit through gaps significantly smaller than their resting size and to align with flow under high shear conditions. RBCs also have a propensity to reversibly aggregate due to interactions with plasma proteins. Together, these RBC characteristics lead to shear thinning behaviour (Chien 1970) that is highly dependent on the haematocrit (Merrill et al. 1963; Pries et al. 1992), amongst other parameters. In large ‘macrovessels’ (arteries and veins), the RBCs are sufficiently small that their distribution can be considered uniform. Macrohaemodynamic analyses therefore typically treat blood as Newtonian or use shear-dependent non-Newtonian viscosity models. However, microvessels have diameters less than 100 $\upmu$m–200 $\upmu$m, such that the RBCs are of comparable length scales to the vessel dimensions and the distribution of RBCs in the microvasculature is highly heterogeneous. As blood rheology is dependent on local RBC concentration, the rheological environment of microvessels is correspondingly complex.

As deformable particles, RBCs migrate radially away from the vessel walls, which is countered by margination due to collisions with other RBCs. The result is varying spatial distributions of RBC concentration (or haematocrit) across the vessel cross section, with generally lower haematocrit adjacent to the vessel wall (Aarts et al. 1988; Moger et al. 2004). This phenomenon leads to a reduction in haematocrit with tube diameter, known as the Fåhraeus effect. At bifurcations, side branches draw fluid from a region of the parent vessel with lower haematocrit, resulting in a net reduction in haematocrit in the side branch, referred to as the Zweifach–Fung or Bifurcation ‘law’. However, not only the average haematocrit, but also the distribution of RBCs within the side branch is changed, with haematocrit skewed towards higher values at the inner wall of the daughter branch (that closest to the bifurcation point). Although not widely addressed, this phenomenon can be seen in previous studies *in vivo* (Pries et al. 1989; Manjunatha and Singh 2002; Ye et al. 2016), *in vitro* (Sherwood et al. 2014b; Shen et al. 2016) and *in silico* (Balogh and Bagchi 2017; Lykov et al. 2015; Ye et al. 2016; Zhou et al. 2021). Even on vessel scales where the continuum assumption breaks down ($\approx 20\,\upmu\mathrm{m}$ (Cokelet 1999)), the time-averaged haematocrit distribution follows these characteristics, as demonstrated elegantly in a numerical study of a capillary network (Balogh and Bagchi 2018).

Experimental studies of haematocrit distributions have predominantly been carried out in long straight capillary tubes *in vitro* (Alonso et al. 1995; Pries et al. 1992) or selected regions of microvascular networks *in vivo* (Kim et al. 2007; Yalcin et al. 2011), where a large distance between bifurcations allows for a ‘cell-free’ or ‘cell-depleted’ layer to develop at the walls (CFL or CDL, respectively). In practice, even where a CFL has had sufficient distance to form, it will be disrupted at the next bifurcation and will take many vessel diameters to recover (Ye et al. 2016), with high likelihood of another bifurcation occurring before this happens, dependent on the particular microvascular network of interest (Bishop et al. 2001; Luo et al. 2017; Zhou et al. 2021). Notably, even in 1969 Merrill noted:

‘It is surprising that in the literature so much emphasis has been placed on annular layers of clear plasma at the wall of the living vessel in spite of the cinematographic proof that under normal conditions no such layer exists’ (Merrill 1969).

Thus, while there are regions of microvascular networks with cell-free layers (particularly in smaller vessels), they are not present throughout. Furthermore, studies that characterise the CFL often also assume a ‘core’ region (the region of the vessel that is not the CFL/CDL) with uniform haematocrit (Cokelet and Goldsmith 1991; Sharan and Popel 2001; Gould and Linninger 2015; Sriram et al. 2014), although the haematocrit actually varies throughout the vessel (Aarts et al. 1988; Moger et al. 2004; Passos et al. 2019). Hence, we contend that it is more appropriate to consider the CFL as part of a continuous distribution of haematocrit that varies throughout a microvascular network. Experimental studies of haematocrit distribution *in vitro* (Aarts et al. 1988; Moger et al. 2004) and *in vivo* (Manjunatha and Singh 2002) provide evidence to support this approach. It should be noted that this paradigm assumes a time-averaged haematocrit value, such that instantaneous fluctuations caused by individual RBCs are smoothed out and thus not directly accounted for, and may break down for vessel scales smaller than $20\,\upmu \mathrm{m}$ Cokelet (1999). Although the CFL (due to RBC migration) is not present throughout the microvasculature, there are reasons why the time-averaged haematocrit in a region immediately next to the walls would be significantly reduced. Firstly, due to the finite size of RBCs, there is a region near the wall where the average haematocrit approaches zero reduced due to exclusion of RBC barycentres (Secomb 2017). Secondly, *in vivo* the endothelial surface layer (ESL) significantly inhibits flow in a region of $\approx 1\,\upmu \mathrm{m}$ adjacent to the vessel wall (Pries 2005).

**Fig. 1:**
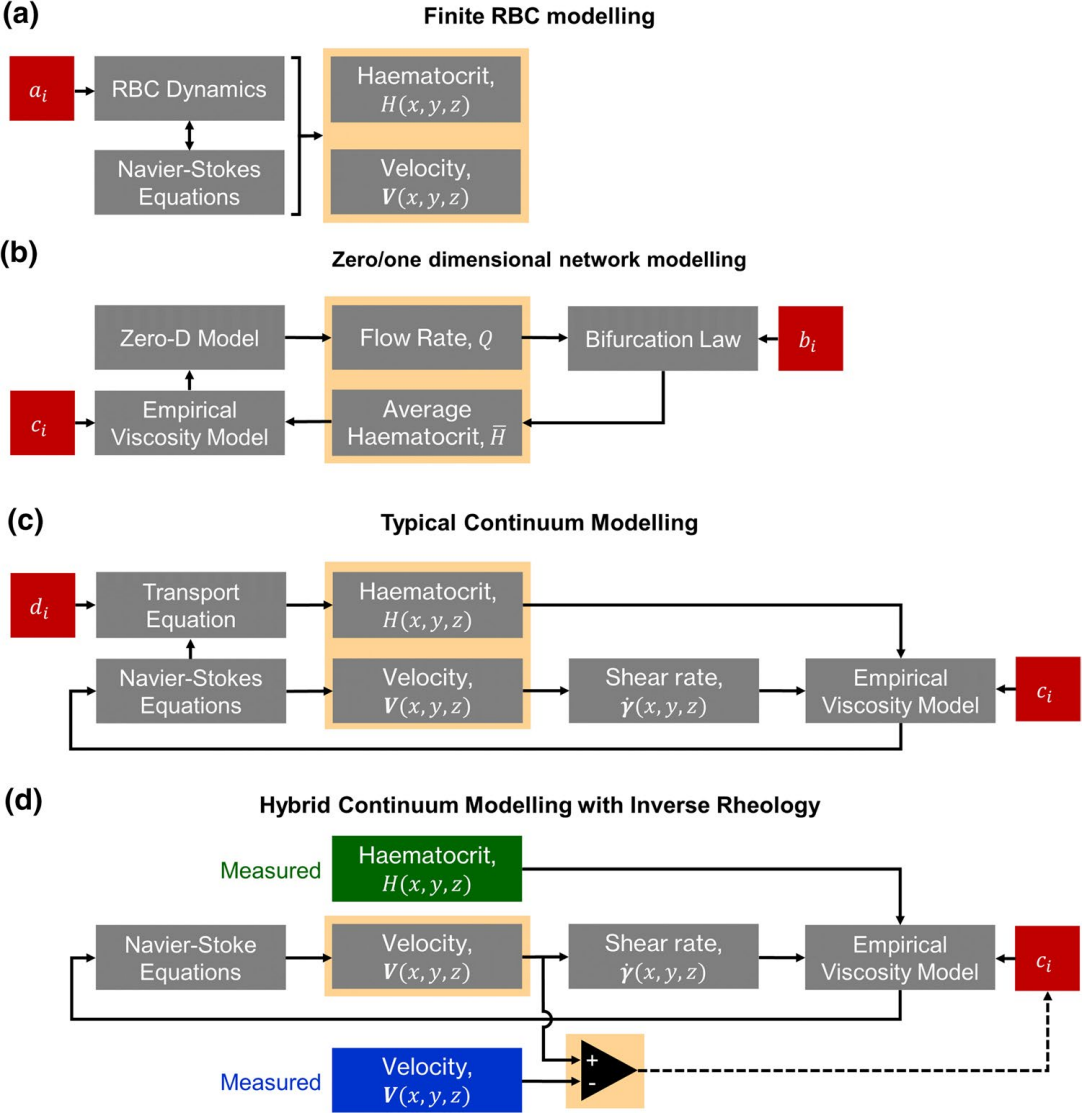
Block diagrams of different approaches to modelling microvascular blood flow. Shaded orange regions indicate outputs from the model. **a** In finite RBC models, each RBC is modelled independently along with a continuum model of the plasma. Empirical parameters are used to define the RBC dynamics. **b** For zero- and one-dimensional network modelling, the network is modelled as a series of interconnected hydrodynamic resistances, with each dependent on local haematocrit via an empirical viscosity model. **c** The typical continuum approach has empirical parameters $c_i$ and $d_i$ as inputs to the Navier–Stokes and transport equations, respectively, to estimate velocity and haematocrit distributions. **d** The hybrid continuum modelling approach uses experimental measurements of haematocrit (green) and intrinsic estimates of shear rate from the Navier–Stokes equations as inputs to an empirical viscosity model with parameters $c_i$ as inputs. By direct comparison with experimental measurements of the velocity (blue), an error minimisation approach is used to optimise $c_i$, which can then be applied to additional data sets. This process does not provide haematocrit as an output, but does provide an estimate of the accuracy of the model

A large number of numerical approaches have been proposed to model the complex fluid environment of the microvasculature, all of which depend on empirical input parameters. In the most detailed ‘Finite RBC models’, RBC dynamics are simulated using techniques such as lattice Boltzmann, immersed boundary or dissipative particle dynamics (see reviews from, for example, Arciero et al. 2017; Freund 2014; Gompper and Fedosov 2016; Omori et al. 2014). These models are dependent on various input parameters ($a_i$, Fig. 1a) describing RBC deformation as well as RBC aggregation (via imposition of attractive forces between RBCs). Through coupling with Navier–Stokes analysis of the suspending medium (plasma, typically treated as Newtonian), the location, velocity and orientation of RBCs can be calculated. Such modelling approaches are increasing in elegance and efficiency, but are fundamentally restricted by the large number of RBCs, even at microvessel scales. A vessel section such as investigated in this study would contain 7000 RBC at a given moment in time, with a turnover rate of 1 s. As a result, even with supercomputing, finite RBC modelling is limited by computational expense and hence typically applied to simulation in two dimensions (Ye et al. 2016), at vessel scales below $\approx 30\,\upmu \mathrm{m}$ in three dimensions (Balogh and Bagchi 2017; Freund and Vermot 2014; Lykov et al. 2015) or at low haematocrits (Zhou et al. 2021). For larger vessels, finite RBC approaches have predominantly been used to model long, straight vessel sections using periodic boundary conditions for efficiency (Lei et al. 2013).

At the other end of the scale, modelling of microvascular networks tends to use simplified phase separation models (Pries et al. 1989; Rasmussen et al. 2018), with empirical relationships for distribution of average haematocrit at bifurcations (Zweifach–Fung ‘law’, parameters $b_i$, Fig. 1b) and haematocrit and vessel diameter-dependent viscosity ($c_i$, Fig. 1b). The parameters $b_i$ and $c_i$ are typically based on a few *in vivo* animal studies in accessible organs and thus do not account for differences between organs and mechanical properties of RBCs in different species. Such models are extremely computationally efficient and able to reproduce network-scale parameters, but are not able to recapitulate local distributions (Rasmussen et al. 2018).

Between these two extremes lies a range of continuum modelling approaches, wherein local viscosity is dependent on both local shear rate and local haematocrit (Chebbi 2018; Jung and Hassanein 2008; Mansour et al. 2010; Schenkel and Halliday 2020; Xu and Kleinstreuer 2019). These models can be solved using established finite volume solvers and require (1) momentum equations (Navier–Stokes) for the mixture or mixture components (plasma and RBCs), (2) a transport equation to describe the RBC behaviour (advection, shear-induced diffusion, migration away from walls and down shear and viscosity gradients) and (3) a viscosity model to couple the local viscosity to the local shear rate and haematocrit (Fig. 1c). The continuum approach is therefore significantly more computationally efficient than finite RBC modelling approaches, providing the possibility of scaling to whole microvascular networks without the need for supercomputing. However, difficulties arise in selecting

(i) appropriate transport equations and corresponding parameters, $d_i$, to account for migration and margination of RBCs,
(ii) an empirical viscosity model and corresponding parameters $c_i$, to account for nonlinear haematocrit and shear dependence, typically derived from data in constant shear rheometers.

More complex models have also been proposed to incorporate, for example, thixotropy and viscoelasticity (Giannokostas et al. 2021) or aggregate distribution (Kaliviotis et al. 2018; Tsimouri et al. 2018), but these require additional parameters.

Irrespective of the modelling technique chosen, the reliance on typically 5–10 parameters and a range of available phenomenological models make the problem ill-defined, and proper validation requires high-quality experimental data, which is scarce. Accordingly, validation of both finite RBC and continuum models has generally been limited to straight tubes, analytical solutions or constant shear rheometry data. As such, it remains unclear how well any of these models recapitulate blood flow behaviour in branching networks on the scale of the microvasculature.

In the present study, we bypass some of the difficulties associated with the dependence on the number of interacting parameters in the continuum approach, using a hybrid experimental–numerical method that combines data measured in a microchannel model with 3D continuum computational fluid dynamics (CFD). This approach offers two main advantages. Firstly, rather than modelling the distribution of RBCs using a transport equation, we measure it experimentally (Passos et al. 2019; Sherwood et al. 2014a, b) and use this as an input to a continuum model using CFD (Fig. 1d). This removes the parameters $d_i$, significantly reducing the parameter space. Secondly, by comparison with experimentally measured velocity data, it is possible to optimise the parameters $c_i$ in order to obtain a numerical model that can predict haemodynamics as measured experimentally. We term this optimisation approach ‘inverse rheology’ (Bandulasena et al. 2011), the CFD equivalent of inverse finite element modelling, an established technique used to similar effect in solid mechanics modelling.

We use previously published experimental data (Sherwood et al. 2014a) that comprises haematocrit distribution and velocity data in a sequentially bifurcating microchannel with a square cross section at a range of input flow rates and flow ratios between branches. The modelling assumes a mixture and uses the semi-empirical Quemada viscosity model, perhaps the most widely used viscosity model that incorporates both haematocrit and shear dependence (Cokelet 1987; Das et al. 1998; Popel and Enden 1993; Sriram et al. 2014). Initial parameters $c_i$ are derived from previously published data from a constant shear rheometry system (Cokelet 1987). We then carry out the inverse rheology approach on two aspects of the model: (1) the parameters $c_i$ and (2) the thickness of the exclusion layer that reduces haematocrit in the region immediately next to the wall (where experimental data could not be acquired due to diffraction). Using a subset of eight experimental cases, we identify the parameters $c_i$ and the thickness of exclusion layer that minimise the error between experimental and numerical velocity profiles. Finally, we apply the model to all 38 experimental cases with different flow conditions and evaluate the relative importance of shear thinning *vs* the exclusion layer, and haematocrit *vs* shear dependence of the viscosity model.

## Methods

### Experimental methods

The inverse rheology approach is built on experimental data published previously Sherwood et al. (2014a), and detailed methods can be found therein. A brief description is provided in the following section.

#### Perfusion set-up and parameters

**Fig. 2:**
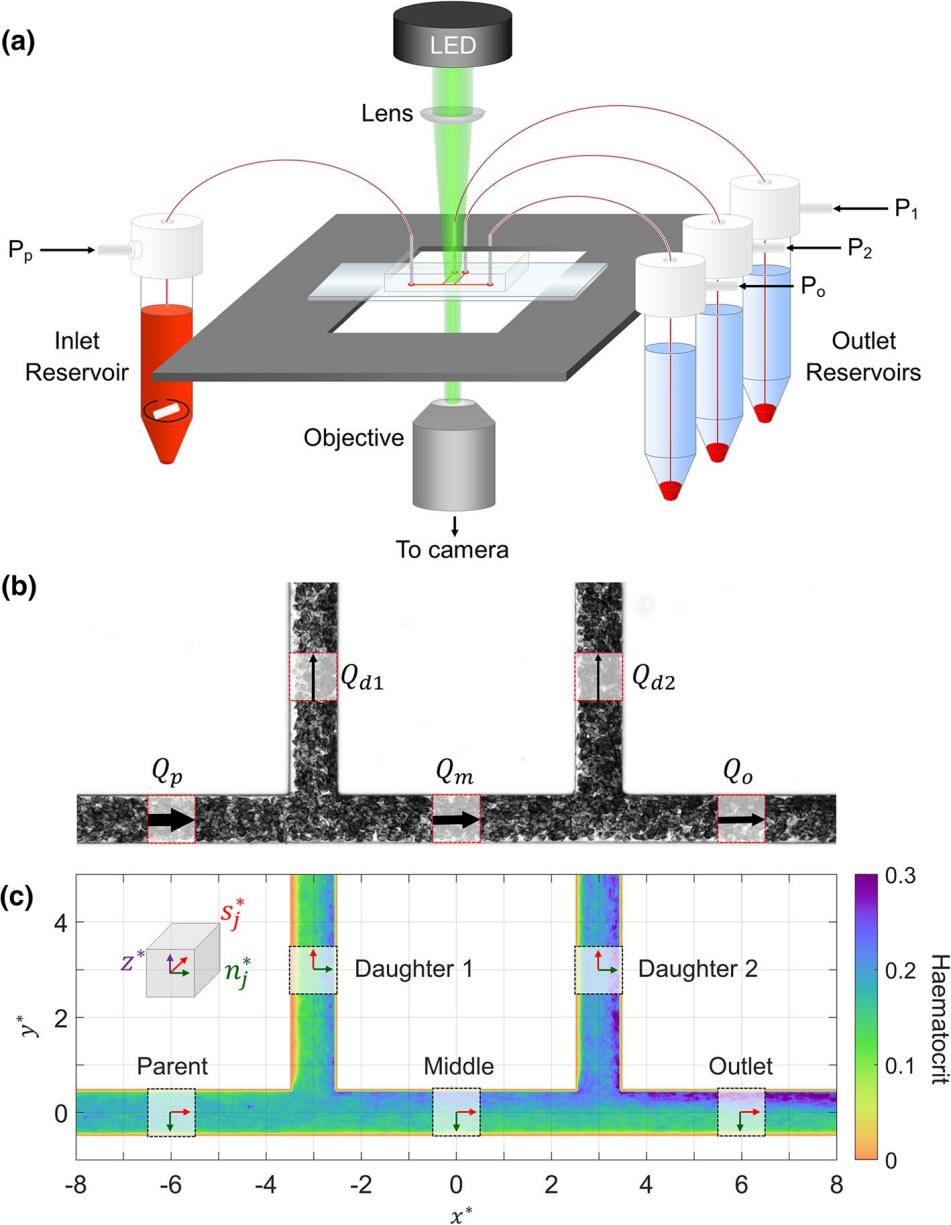
**a** Experimental set-up. Blood is perfused through the microchannel using a microfluidic pressure controller. The blood in the inlet reservoir is mixed using a magnetic stir bar to counteract RBC sedimentation. A high-power LED is focussed on the region of interest in the channel, with transmitted light collected via an objective into a PIV camera. The microchannel features a sequential bifurcation with flow entering from the parent branch and exiting via daughter 1, daughter 2 or outlet branches. **b** Channel dimensions and flow rates. Each grid square represents $50\,\upmu \mathrm{m}$. Arrows indicate flow direction, with thickness representing relative proportion of inlet flow. Dashed red lines indicate regions of interest (ROI) in each branch **c** Global and local coordinate systems, branch names and sample haematocrit distribution. Axes $x^*$ and $y^*$ provide scales in terms of channel widths

The experimental set-up (Fig. 2a) used a high-power green LED (Dantec Dynamics) to illuminate a blood sample passing through a microchannel mounted on an inverted microscope (Leica DM-ILM, Germany) using a 10X objective ($\mathrm{NA}=0.25$). A Hamamatsu C8484-05C (Hamamatsu, Japan) PIV camera was used to capture image pairs, focused on the central plane of the channel at 3 Hz. The LED was pulsed once within each exposure of an image pair, with a pulse width of 0.1 ms. The delay *dt* between pulses ranged from 1 to 8 ms, depending on the flow rates in the system. All triggering and acquisition were carried out using LabVIEW (National Instruments).

The microchannel master was fabricated using deep resistive ion etching (DRIE) of S1818 photoresist, and channels were cast in polydimethylsiloxane (PDMS) and bonded onto glass slides using standard approaches. The channels had a square cross section with a width of $w=50\,\upmu \mathrm{m}$ and featured two side branches at 90 degrees to the main channel, with 5*w* between them (Fig. 2b, c). Pressures at all four branches were regulated using a pressure control system (MFCS-8C: Fluigent, France). For each acquisition, a uniform distribution of RBCs was first established by mixing the inlet reservoir with a magnetic stir bar and perfusing the channel at a high pressure drop. The flow rate was then dropped to a target value and held there for 20 s for RBC aggregation to reach a steady state prior to acquisition. Sixty image pairs were then acquired before returning the pressure to a high value ($\approx 20\,\mathrm{kPa}$). Without this process, the haematocrit delivered to the channel would vary greatly due to sedimentation and aggregation of RBCs throughout the fluid system. Despite the high-resolution microfluidic pressure control system used, it was not possible to repeatedly get the same flow distribution and flow rates in the parent branch. Instead, a ‘scattershot’ approach was taken with 38 cases comprising different input flow rates (0.1–1.2 $\upmu$l/min) and flow ratios, $Q^*$, defined as the proportion of the flow entering a given branch of a bifurcation.

#### Blood samples

The study was approved by the South East London Research Ethics Committee (Reference 10/H0804/21), and informed written consent was obtained from volunteers. Blood was sampled from a healthy individual via venepuncture and mixed with 1.8 mg/ml EDTA to inhibit coagulation. RBCs were separated by centrifugation from the plasma and buffy coat, which were aspirated and replaced with phosphate-buffered saline (PBS). This process was repeated twice, then the RBCs were resuspended in PBS supplemented with Dextran 2000 at 5 mg/ml. This induced a physiological level of aggregation, independent of the plasma protein levels of the volunteer. All samples were used within 4 h of extraction or were refrigerated and used within 3 h of returning to room temperature.

#### Velocity measurement

Blood velocity was calculated using particle image velocimetry (PIV) processing of the RBC images using JPIV (https://eguvep.github.io/jpiv/), an open licence PIV software package. Ensemble (or correlation) averaging of 60 image pairs acquired over 20 s was used for each case. Each branch of the bifurcation was cropped to transverse width *w* (84 pixels) and axial length 4.5*w* and analysed with interrogation windows of $32 \times 4$ pixels (axial $\times$ transverse), with vector spacing of $8 \times 2$ pixels. A region of 6 pixels adjacent to the wall was not usable due to diffraction at the channel edge, so the first vector was located 8 pixels ($4.8\,\upmu \mathrm{m}$) from the wall.

A *w* length ROI was selected for each branch (Fig. 2b, c). Spurious vectors were identified as having velocities further than three median absolute deviations from the median for a given transverse vector location. This one-dimensional approach was used instead of the more common normalised median test (Westerweel and Scarano 2005) to avoid transverse smoothing of the velocity profile and resulted in removal of 3% of vectors on average. The mean and two standard deviations of the velocity at each transverse location were then taken to represent the velocity profile for a given ROI. Finally, the velocity magnitude was scaled by a factor of 1.5, to account for the fact that the PIV analysis would be ‘depth saturated’ (Poelma et al. 2012), i.e. the depth of correlation (DOC) is greater than the channel depth, leading to an underestimate of the velocity in the central plane of the channel. As normalised profiles are considered, the main uncertainty introduced by this adjustment is a potential systematic error on the absolute values of shear strain rate, which is implicitly addressed in the inverse rheology process.

#### Concentration measurement

**Fig. 3:**
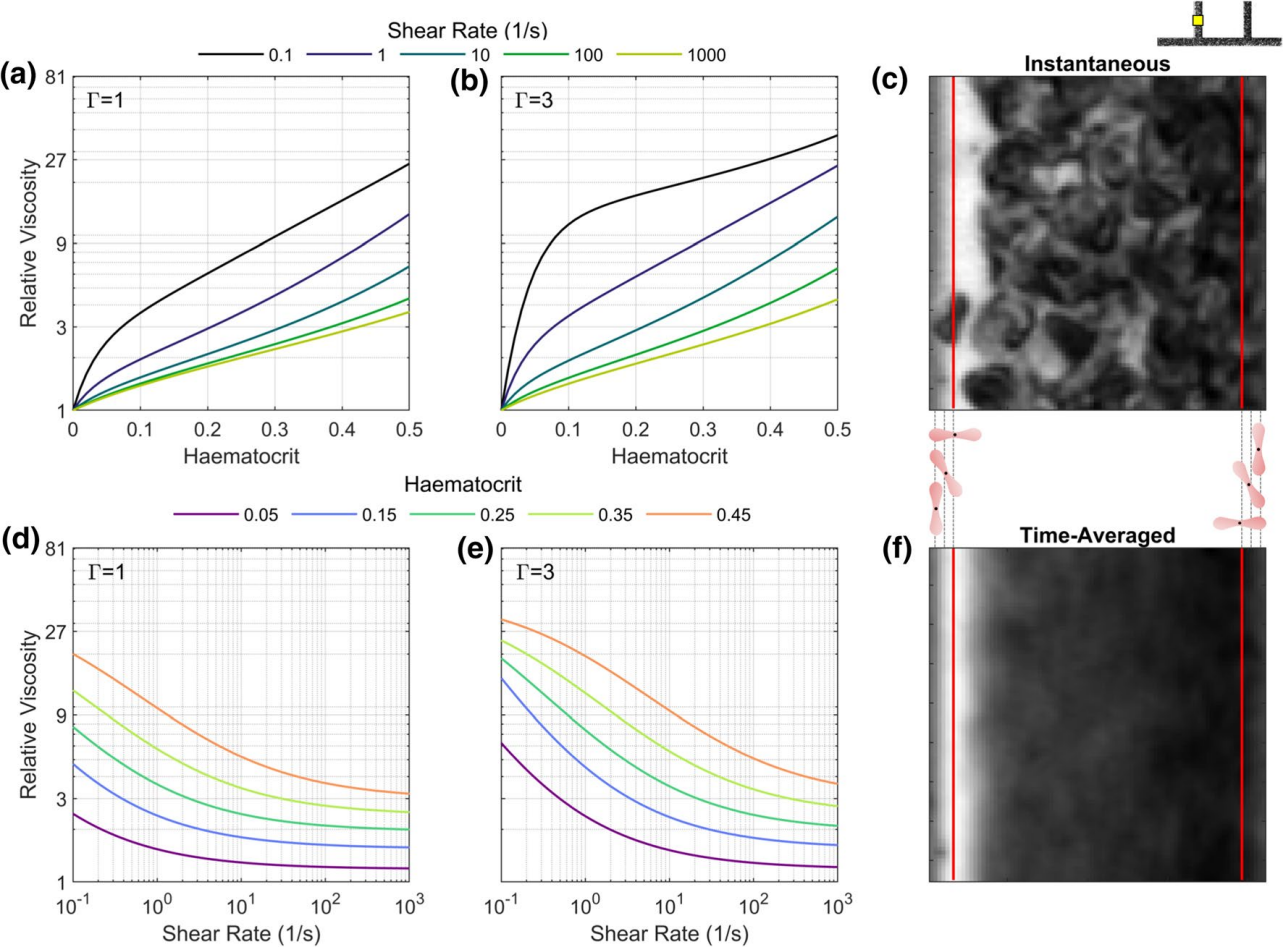
Effects of the viscosity model modifications investigated using the inverse rheology approach. **a, b** Quemada viscosity as function of haematocrit for a range of shear rates with $\varGamma =1$ (original data from Cokelet 1987) and $\varGamma =3$ (final selected value). **d, e** Quemada viscosity as function of shear rate for a range of haematocrits with $\varGamma =1$ and $\varGamma =3$. **c, f** Instantaneous and time-averaged images of RBCs, with scale diagrams of RBC orientation. Black dots indicate RBC barycentre, and dashed lines indicate exclusion zones 2, 4 and 6 pixels from the wall, corresponding to 1.2, 2.4 and 3.6 $\upmu$m

The average intensity at each pixel location was calculated from the 120 images used in each PIV analysis. This was used to calculate the depth-averaged concentration via a calibration procedure described previously (Sherwood et al. 2014a). Briefly, an intensity–haematocrit curve was generated by perfusing solutions of RBCs in Dextran/PBS solution at various haematocrits through the microchannel, with empirical amendments for the Fåhraeus effect (Pries et al. 1990) and red cell screening (Gaehtgens et al. 1978) to estimate the channel haematocrit. Due to the diffraction region of 6 pixels next to the vessel wall (red lines in Fig. 3c, f), it was not possible to accurately resolve the haematocrit at the wall. Even without this imaging artefact, the effective size of the ‘exclusion layer’ ($\xi$) adjacent to the vessel wall using the continuum approach would require attention. As indicated in Fig. 3, the size of the exclusion region cannot be determined directly because of the different orientations of RBCs, as well as their deformability (not visualised).

We therefore modelled the exclusion layer during the inverse process as a region 1–5 pixels from the wall ($0.6\,\upmu \mathrm{m}/{\text {pixel}}$). To estimate the haematocrit in the 6 pixels adjacent to each wall, a second-order polynomial was fit between the first three valid pixels (7, 8 and 9 pixels away from the wall) and a value of zero at the imposed exclusion layer thickness.

#### Local coordinate systems

A local coordinate system ($s_j^*$, $n_j^*$, $z^*$) was defined in each branch (Fig. 2c), with the ${}^*$ indicating normalisation by *w*. In the parent, middle and outlet branches, the axial coordinate $s_j^*$ was parallel to $x^*$, and the transverse coordinate $n_j^*$ was antiparallel to $y^*$. In the daughter branches, $s_j^*$ was parallel to $y^*$, and $n_j^*$ was parallel to $x^*$. Local origins were defined in the centre of each ROI shown in Fig. 2c. The $z^*$ axis was the same for all coordinate systems.

#### Extrapolation into three dimensions

To obtain a 3D model of the haematocrit distribution, and to appropriately calculate branch flow rates for normalisation of the velocity profiles, the velocity and haematocrit data were extrapolated into three dimensions. This was done on the basis that the velocity and haematocrit in the parent branch would be fully developed (having followed a straight section of $\approx 200w$) and thereby exhibit fourfold symmetry. This implies that for an arbitrary parameter $\psi (s_j^*,n_j^*,z^*)$ in the parent branch (subscript *p*):

1 $$\begin{aligned} \psi \left( s_p^*,n_p^*,0\right)&= \psi \left( s_p^*,-n_p^*,0\right)\\ &= \psi \left( s_p^*,0,n_p^* \right) =\psi \left( s_p^*,0,-n_p^*\right) \end{aligned}$$

Furthermore, based on the fact that the length and timescales of the system are insufficient for RBC sedimentation to have an impact (Alonso et al. 1995; Sherwood et al. 2014b) and that the geometry is uniform in $z^*$, it is reasonable to assume that the shape of the distribution in the $z^*$ direction does not change, and thus, $\psi (s_p^*,n_p^*,0)$ can be used to extrapolate into three dimensions as follows.

*Haematocrit. * The haematocrit measurements are based on light transmitted through the sample and are therefore depth-averaged, hence for a given branch *j*

2 $$\begin{aligned} H\left( s_j^*,n_j^*,z^* \right) =H\left( s_j^*,n_j^*,0\right) \frac{H_p \left( s_j^*,z^*,0\right) }{\overline{H_p}} \end{aligned}$$

To avoid imposing asymmetries in $z^*$, $H_p$ is replaced by a symmetric profile of the form

3 $$\begin{aligned} H_{\mathrm{sym}}\left( s_p^*,n_p^*,0\right) =H_{p,\mathrm{max}} \left( \frac{\text {cosh}\left( 0.5\right) ^{\beta _h}-\text {cosh}\left( \left| n_p^*\right| \right) }{\text {cosh}\left( 0.5\right) ^{\beta _h}-1}\right) \end{aligned}$$

with $\beta _h$ determining the haematocrit profile ‘bluntness’ and $H_{p,\mathrm{max}}$ being the maximum haematocrit. Equation (3) was fitted to $H\left( s_p^*,\pm n_p^*,0\right)$.

To incorporate the exclusion layer without modifying the average haematocrit, the values of $H_{\mathrm{sym}}$ for $\left| n_p^*\right| >w/2-\xi$ are set to zero prior to calculating $\overline{H_p}$.

*Velocity.* The velocity profile calculated with PIV is the value at the central plane (having accounted for depth of correlation effects); hence, the 3D velocity distribution in a given branch *j* is given by

4 $$\begin{aligned} u\left( s_j^*, n_j^*, z^*\right) =u\left( s_j^*,n_j^*,0\right) \frac{u_p \left( s_p^*,z^*,0\right) }{\text {max}(u_p)} \end{aligned}$$

As with the haematocrit, to avoid imposing asymmetries in $z^*$, $u_p$ is replaced by a symmetric profile of the form

5 $$\begin{aligned} u_{\mathrm{sym}} \left( s_p^*,n_p^*,0\right) =u_{p,\mathrm{max}} \left( \frac{\text {cosh}\left( 0.5\right) ^{\beta _v}-\text {cosh}\left( \left| n_p^*\right| \right) }{\text {cosh}\left( 0.5\right) ^{\beta _v}-1}\right) \end{aligned}$$

with $\beta _v$ determining the velocity profile ‘bluntness’ and $u_{p,\mathrm{max}}$ being the maximum velocity. Equation (5) was fitted to $u\left( s_p^*,\pm n_p^*,0\right)$.

#### Calculation of flow rate and conservation of mass

The flow rate in a given branch *j* at $s_j^*$ is given by

6 $$\begin{aligned} Q_j=\int _{-w/2}^{w/2}\int _{-w/2}^{w/2}u \left( s_j^*,n_j^*,z^*\right) dn_j^* dz^* \end{aligned}$$

However, the experimental velocity data are not defined for the 6 pixel ($4.8\,\upmu \mathrm{m}$) region adjacent each channel wall. In order to implement Eq. (6), the velocity in these regions must first be estimated. Whether the RBC velocity at the wall should equal zero or a finite value due to rolling of RBCs along the wall is an unresolved question (Roman et al. 2016). However, as the RBC concentration at the wall is set to zero and the suspending medium is expected to follow the no-slip condition, a zero velocity at the wall is imposed in the present study. Equation (7) is a modification of Eq. (5) that captures zero wall velocity and enables asymmetry:

7 $$\begin{aligned}&u_{\mathrm{asym}} \left( s_j^*,n_j^*,0\right) =\alpha _1\left( 1+\alpha _2n_j^*+\alpha _3{n_j^*}^2\right) \nonumber \\&\quad \left( \frac{\text {cosh}\left( 0.5\right) ^{\beta _a}-\text {cosh}\left( \left| n_p^*\right| \right) }{\text {cosh}\left( 0.5\right) ^{\beta _a}-1}\right) \end{aligned}$$

where $\alpha _1$ is the maximum velocity when $\alpha _2=\alpha _3=0$. The $\alpha _2$ term allows for asymmetry in the profile and $\alpha _3$ provides an additional degree of freedom to achieve a tight fit with the experimental data. The value of $\beta _a$ relates to the bluntness of the velocity profile.

This approach uses all of the measured velocity values and has no free user parameters, in contrast to using, for example, a polynomial extrapolation that would be dependent on the selection of the number of data points to use and the order of the polynomial.

After Eq. (7) is fit to the experimental data, Eq. (4) is used to generate an ($n_j^*, z^*$) plane velocity profile, upon which Eq. (6) is implemented using trapezoidal integration.

The estimated flow rates were used to compute the error in the velocity measurements. The errors in the first and second bifurcations are given by:

8 $$\begin{aligned}&e_{Q,1}=\frac{Q_p-Q_{d1}-Q_m}{Q_p} \times 100\% \end{aligned}$$

9 $$\begin{aligned}&e_{Q,2}=\frac{Q_m-Q_{d2}-Q_o}{Q_m} \times 100\% \end{aligned}$$

where the subscripts refer to the branches (Fig. 2). Errors across the whole system are given by

10 $$\begin{aligned} e_{Q,\mathrm{tot}}=\frac{Q_p-Q_{d1}-Q_{d2}-Q_o}{Q_p} \times 100\% \end{aligned}$$

Note that this calculation assumes that at the domain boundaries, blood can be treated as a single-phase mixture with velocity that is reliably tracked by the RBCs.

To estimate the overall experimental error, the RBC volumetric flux calculated at $s_j^*$ is used. This is given by

11 $$\begin{aligned} F_j=\int _{-w/2}^{w/2}\int _{-w/2}^{w/2}u\left( s_j^*,n_j^*,z^*\right) H\left( s_j^*,n_j^*,z^*\right) dn_j^* dz^* \end{aligned}$$

Equations (8)–(10) were applied with *F* replacing *Q*. Table 1 summarises the statistics of these data. The conservation of mass errors indicate that each bifurcation is associated with a small negative offset ($-3$% and $-4$% in Bifurcations 1 and 2, respectively), with a larger discrepancy when evaluating the error across all external branches of $-7$% (see Table 1). Supplemental Figure S1 plots these error terms against flow ratio and parent branch flow rate. The average RBC flux errors are the same in Bifurcation 1 ($-3$%), but larger in Bifurcation 2 ($-11$%) with an average of $-12$% for the external branches. Poelma et al. (2012) reported that the underestimation of RBC velocity scaled with absolute velocity. Hence, to investigate whether this could be the cause of the offset, we numerically identified that raising all measured velocities to the power 1.066 before calculating $e_Q$ eliminated the average errors in mass conservation. Applying this small amendment in the flux calculation reduced the errors in the RBC flux to $-5$% for all external branches (Figure S1, Table 1), indicating some contribution to the error from either the haematocrit measurements or the assumption of a single-phase continuum mixture with no relative velocity between phases. Nonetheless, given the multiple processing steps and extrapolations required to calculate these flows and fluxes, we consider these uncertainties to be acceptably small for the present analysis.

**Table 1:** Errors in conservation of mass and RBC flux

	Bif. 1	Bif. 2	Total
	$e_{Q,1}$	$e_{Q,2}$	$e_{Q,\mathrm{tot}}$
Flow rate	− 3 ± 3	− 4 ± 2	− 7 ± 2
RBC flux	−3 ± 5	− 11 ± 4	− 12 ± 2
Flow rate amended	0 ± 2	0 ± 2	0 ± 2
RBC flux amended	0 ± 4	− 7 ± 3	− 5 ± 3

Numbers are mean ± two standard deviations. Amendment involves raising the measured velocities to the power 1.066, which empirically eliminates the average error in flow calculation, but not flux

#### Skewness indices

Asymmetry of velocity and haematocrit distributions plays a key role in determining the flow characteristics in all branches except the parent branch. It is therefore useful to quantify the extent of this asymmetry in each branch to provide context for the optimisation process described below. The skewness of the haematocrit distribution is characterised according to

12 $$\begin{aligned} S_{H,j}=\frac{\int _{0}^{0.5}H\left( s_j^*,n_j^*,0\right) dn_j^*}{\int _{-0.5}^{0.5}H\left( s_j^*,n_j^*,0\right) dn_j^*}-0.5 \end{aligned}$$

similarly to our previous study (Sherwood et al. 2014a), but without taking the absolute value as was done therein. An $S_{H,j}$ value of 0.1, for example, would indicate that the positive $n_j^*$ side of the axis has 10% more RBCs than a symmetric profile or equivalently 20% more than the negative $n_j^*$ side of the axis. These calculations are carried out for $\xi =3$ pixels (see Sect. 2.2.3). The skewness of the velocity profile is taken as the value of $n_j^*$ corresponding to the location of maximum velocity (carried out on the fit to Eq. (7) evaluated at 1001 locations across the channel width):

13 $$\begin{aligned} S_{u,j}=n_j^*\left( \text {max}\left[ u_{\mathrm{asym}}\left( s_j^*,n_j^*,0\right) \right] \right) \end{aligned}$$

### Numerical methods

#### Viscosity model

In general, an empirical model is required to characterise the function $\mu \left( H,{\dot{\gamma }}\right)$ (parameters $c_i$ in Fig. 1). There are many viscosity models that can account for the effects of both shear rate and RBC concentration on viscosity (see, for example, Hund et al. 2017; Sousa et al. 2016; Yilmaz and Gundogdu 2008 for detailed summaries). These models are typically fit to data obtained from bulk rheology measurements, to characterise the parameters $c_i$ (Fig. 1b–d). For the present study, we focus on the Quemada model (Dufaux et al. 1980), a semi-empirical model derived for planar flow that has been widely used in blood flow modelling (Buchanan et al. 2000; Cokelet 1987; Das et al. 1998; Mansour et al. 2010; Marcinkowska-Gapińska et al. 2007; Popel and Enden 1993; Schenkel and Halliday 2020), although the method described here is applicable to any model. The Quemada model describes the viscosity according to

14 $$\begin{aligned} \mu =\frac{\mu _p}{\left( 1-0.5kH\right) ^2} \end{aligned}$$

where $\mu _p$ is the plasma viscosity and *k* is referred to as the intrinsic viscosity, which is given by:

15 $$\begin{aligned} k=\frac{k_0+k_\infty \sqrt{\dfrac{\left| {\dot{\gamma }} \right| }{ {\dot{\gamma }}_c}}}{1+\sqrt{\dfrac{\left| {\dot{\gamma }} \right| }{ {\dot{\gamma }}_c}}} \end{aligned}$$

with $k_0$ as the intrinsic viscosity at zero shear rate and $k_\infty$ as the intrinsic viscosity at infinite shear rate. The critical shear rate ${\dot{\gamma }}_c$ acts as a scaling factor for the shear rate. Cokelet (1987) published experimental data describing $k_0$, $k_\infty$ and ${\dot{\gamma }}_c$ as functions of haematocrit. Haematocrits of up to 0.7 were used, and fourth-order polynomials in the log domain were used to fit the data. Subsequent studies have noted the discontinuities caused by this fitting approach and have proposed amendments to the fit (Das et al. 1998; Schenkel and Halliday 2020). In the present study, to reduce the number of variables and focus on haematocrits of relevance to microvascular flow, we applied new fits to the original data, as described in Table 2. The resulting relationships for $k_0\left( H\right)$, $k_\infty \left( H\right)$ and ${\dot{\gamma }}_c\left( H\right)$ are shown in Figure S2 and compared to those reported by Cokelet (1987). The relative viscosity curves according to Eqs. (14) and (15) are shown in Fig. 3a, d, at a range of shear rates and haematocrits. The relative viscosity increases approximately exponentially with *H* for $H>0.1$ (Fig. 3a). The viscosity is also sensitive to shear rate, particularly at shear rates below $\approx 10\,\mathrm{s}^{-1}$ (Fig. 3d). For comparison, note that a power law model would be a straight line on these axes.

**Table 2:** Alternative fitting parameters for data from Cokelet (1987) that halves the number of degrees of freedom

$k_0$	$k_\infty$	${\dot{\gamma }}_c$
$\text {ln} \left( k_0\right) =\frac{p_1}{p_2+H}$	$\text {ln}\left( k_\infty \right) =\frac{p_3}{p_4+H}$	$\text {ln}\left( {\dot{\gamma }}_c\right) =p_5H+p_6$
$p_1=0.833$	$p_3=0.532$	$p_5=11.9$
$p_2=0.177$	$p_4=0.399$	$p_6=-4.48$

#### Computational fluid dynamics approach

Computational modelling was carried out using ANSYS CFX. A steady simulation was carried out for each experimental case. The measured haematocrit distributions were mapped into three dimensions as described above and imported into CFX. All branches in the domain were extended by five branch widths to eliminate boundary effects, with the haematocrit in the extended branches being the axial average over the outermost *w*/2. The parent branch velocity was set to the average value calculated from the experimental data, ${\overline{u}}_p$, and the outlet pressure was set to zero. To distribute the uncertainties in flow rate between branches, the flow ratios were calculated as follows:

16 $$\begin{aligned} Q_1^*=\frac{Q_{d1}+\left( Q_p-Q_m\right) }{2Q_p} \end{aligned}$$

17 $$\begin{aligned} Q_2^*=\frac{Q_{d2}+\left( Q_m-Q_o\right) }{2Q_m} \end{aligned}$$

The boundary condition at the outlets of the daughter branches were set to an average velocity, given by ${\overline{u}}_{d1}={\overline{u}}_p Q_1^*$ in daughter Branch 1 and ${\overline{u}}_{d2}={\overline{u}}_p \left( 1-Q_1^*\right) Q_2^*$ in daughter Branch 2. The shear rate in the viscosity model was set intrinsically, with haematocrit imposed extrinsically. An RMS residual target of $10^{-5}$ was used to determine convergence. The ANSYS CFX high-resolution differencing scheme was implemented, which maximises a blend factor between upwind and second-order differencing schemes, while keeping the solution bounded.

An unstructured tetrahedral mesh was generated in ANSYS Workbench using in-built adaptive sizing algorithms. The reference size was selected as an integer multiple of the pixel resolution ($0.6\,\upmu \mathrm{m}$). Wall resolution was improved via 7 ‘inflation layers’, with a first layer height half the reference size and a growth rate of 1.2.

Mesh sensitivity analysis was carried out on a sample case, using 6 different mesh sizes ranging from $\approx 140k$ to $>6M$ elements (see Table S1). The RMS deviation of the velocity profile for each mesh relative to the finest mesh in each branch was calculated and used to characterise the effect of further refinements (Figure S3a). Each refinement reduced the error, although differences were small. The time to run the simulation was also considered for each mesh (Figure S3b) and compared to the time to load the experimental data ($\approx 6$ min). A mesh with a reference size of $1.2\,\upmu \mathrm{m}$, comprising 2.4*M* elements was selected. The average RMS deviation between the velocity profiles across all branches for this mesh and the finest mesh was 0.31%. The selected mesh took 40% less time to run than the finest mesh at $<12$ min including loading the mapped haematocrit data ($\approx 6$ min) on a standard desktop machine.

#### Inverse rheometry and paradigms

For the inverse rheometry process, a subset of 8 cases was selected that represented a wide range of input parent branch velocities (${\overline{u}}_p=0.8-7.9\,\mathrm{mm/s}$) and flow ratios ($Q^*=0.16-0.64$) (see Table 3 for full details). For the present study, we used this process to optimise parameters for (i) the viscosity model and (ii) the size of the exclusion layer, $\xi$.

**Table 3:** Cases selected for the inverse rheology process

Case	${\overline{u}}_p \left( \mathrm{mm/s}\right)$	$Q_1^*$	$Q_2^*$
1	0.81	0.24	0.29
2	1.20	0.16	0.39
3	1.90	0.36	0.46
4	2.22	0.49	0.70
5	3.29	0.25	0.54
6	3.92	0.25	0.37
7	7.10	0.32	0.43
8	7.85	0.33	0.64

*Viscosity model parameters.* Even with the reduced parameter space for the viscosity model (Table 2), six degrees of freedom provide an excessively large number of variables. We therefore focussed on a single modification to the viscosity model: the critical shear rate, ${\dot{\gamma }}_c$. This parameter can be interpreted as the reciprocal of $T_c$, the timescale of the phenomenon that is causing shear-dependent behaviour, which is predominantly RBC aggregation (and disaggregation) and RBC deformability (Cokelet 1987). This interpretation is complicated by the fact that the timescales of aggregation (10–20 s), disaggregation (almost instantaneous) and cell deformation ($\approx 0.06\,\mathrm{s}$) are very different (Cokelet 1980). Furthermore, aggregation timescales are dependent on local shear, the protein content in the plasma (or in this case Dextran concentration in the suspending medium) and the aggregability of the red blood cells. For the original data in whole blood, the parameter $T_c$ decreases from $\approx 10$ min at low haematocrit to 0.2 s at a haematocrit of 0.6, a remarkably large range (Figure S2d). Notably, in the absence of aggregation (having suspended the RBCs in saline), Cokelet (1987) reported a reversed correlation between $T_c$ and haematocrit, with $T_c$ being $\approx 6$ orders of magnitude lower at low haematocrit. We posit therefore that the explicit interpretation of this parameter should be taken only lightly when extrapolating the model to 3D confined blood flows. Finally, it should be noted that the Quemada model was derived for a cone and plate rheometer with constant shear rate and may not be directly translatable to environments with high shear gradients acting over length scales comparable to individual cells. With all this in mind we take an empirical approach to find the optimal viscosity parameters, modifying the intrinsic viscosity term *k* according to

18 $$\begin{aligned} k=\frac{k_0+\frac{k_\infty }{\varGamma }\sqrt{\dfrac{\left| \dot{\gamma } \right| }{ \dot{\gamma }_c}}}{1+\frac{1}{\varGamma }\sqrt{\dfrac{\left| {\dot{\gamma }} \right| }{ {\dot{\gamma }}_c}}} \end{aligned}$$

where $\varGamma =1, 2, 3, 4$ or 5. For a given value of $\varGamma$, the value of ${\dot{\gamma }}_c$ is increased by a factor of $\varGamma ^2$, implying a reduction in the time constant associated with shear dependence (see Figure S2d). This ultimately has the effect of increasing viscosity at lower haematocrit (Fig. 3b) and increasing the sensitivity of the viscosity to shear rate (Fig. 3e) or ‘enhancing’ the shear thinning behaviour.

*Exclusion layer thickness.* As described above, the size of the exclusion layer at the vessel wall remains an open question. To recapitulate, this is not the ‘cell-free layer’ caused by migration of RBCs away from the wall, but a region defined by the geometry of the RBCs, with uncertainty arising due to deformation and orientation of individual RBCs. For each case and value of $\varGamma$, we consider $\xi =1,2,3,4$ or 5 pixels (which corresponds to 0.3, 0.9, 1.5, 2.1 or $2.7\,\upmu \mathrm{m}$ taking the centre of pixel). These dimensions should be interpreted relative to the length scale of RBCs ($\approx 8 \times 2\,\upmu \mathrm{m}$) and the relative location of the cell barycentre, which is dependent on cell orientation (Fig. 3c, f). The effect of increasing $\xi$ will be to introduce an increasingly large lubrication layer at the vessel wall, which like $\varGamma$ would lead to blunter velocity profiles. However, while $\varGamma$ would enhance ‘skewness’ of the measured velocity profiles by enhancing viscosity away from the channel centre, $\xi$ would decrease it by imposing symmetric viscosity conditions at each wall.

**Fig. 4:**
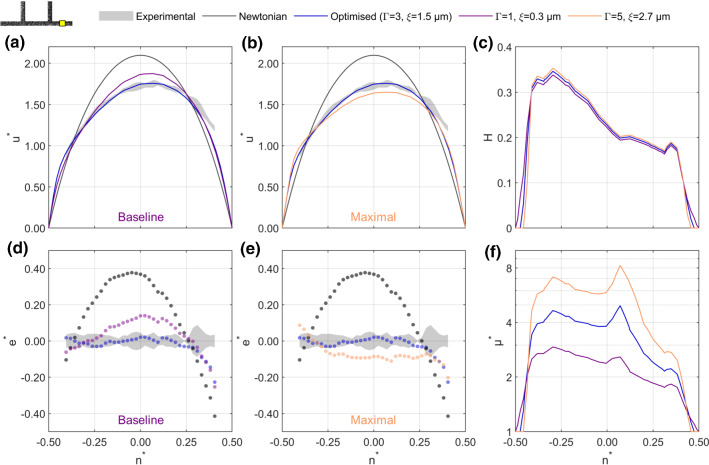
**a, b** Sample normalised velocity profiles in outlet branch for a case with $\overline{u_p}=1.9\,\mathrm{mm/s}$, $Q_1^*=0.33$ and $Q_2^*=0.44$. Profiles using baseline (**a**) and maximal (**b**) modifications applied during the inverse rheology process are compared to final parameters and the Newtonian solution. **d, e** Errors relative to the experimental data for the same cases. **c** Haematocrit profiles with exclusion zones of $\xi =0.3, 1.5$ and $2.7\,\upmu \mathrm{m}$. Note values are scaled to yield equal branch haematocrit. f) Relative viscosity profiles, showing similar shapes but significant difference in magnitude for $\varGamma =1, 3\,\mathrm{and}\,5$

*Evaluation.* In the inverse process, we find single values of $\xi$ and $\varGamma$ that minimise the overall error between the experimental and numerical velocity profiles for all eight cases in Table 3. Quantification of the error is carried out by calculating a single representative value for the error of the simulation in each branch. Velocity profiles were first normalised by the average velocity in that branch to give

19 $$\begin{aligned} u_j^*\left( n_j^*\right) =u\left( n_j^*\right) \frac{w^2}{Q_j} \end{aligned}$$

(see, for example, Fig. 4a). Defining $u_{j,piv}^*$ as $u_j^*$ for the experimental data, a normalised velocity error profile can be calculated according to

20 $$\begin{aligned} e_j^* \left( n_j^*\right) =u_j^*\left( n_j^*\right) -u_{j,piv}^* \left( n_j^*\right) \end{aligned}$$

(see, for example, Fig. 4d). The RMS value of $e_j^*\left( n_j^*\right)$ is then calculated as a measure of how well a given numerical velocity profile matches the experimental velocity profile $u_{j,piv}^*$ over the *N* PIV vectors in the velocity profile:

21 $$\begin{aligned} \text {RMS}\left( e_j^*\right) =\sqrt{\frac{1}{N}\sum {e_j^*}^2} \end{aligned}$$

The magnitude of $\text {RMS}\left( e_j^*\right)$ is not easily interpreted when considered alone; a relative error, $E^*$, is therefore defined as follows

22 $$\begin{aligned} E^*=\frac{\text {RMS}\left( e_j^*\right) }{\text {RMS}\left( e_{j,a}^*\right) } \end{aligned}$$

with $\text {RMS}\left( e_{j,a}^*\right)$ corresponding to $\text {RMS}\left( e_j^*\right)$ for a Newtonian fluid based on the analytical solution (Bruus 2008). The value of $E^*$, varies between 0, indicating a perfect match to experimental data, and 1, indicating the error when a Newtonian fluid is assumed.

*Optimisation process.* The purpose of the inverse process described here is to identify the optimal values of $\varGamma$ and $\xi$ that apply to all experimental cases (using only a small subset), rather than different values for each case. The latter approach would provide reduced errors, but amount to a localised data fitting process. Furthermore, the same blood sample was used for all cases, so a fixed value of $\varGamma$ is necessary for consistency. Instead, identifying globally optimised values of $\varGamma$ and $\xi$ based on a small sub-sample provides a self-consistent model that can be applied to the broader data set. All permutations of each of the eight flow condition cases (Table 3), five values of $\varGamma$ and five values of $\xi$ required 200 simulations. For each branch of each case, $E^*$, was calculated independently. The minimum value of $E^*$ across all branches was then ultimately used to select the final values of $\varGamma$ and $\xi$ to use in the forward modelling stage of the analysis.

#### Forward modelling

Having optimised for $\varGamma$ and $\xi$ we can use the model to investigate questions of interest for the whole data set of 38 experimental cases.

*Relative importance of viscosity model and exclusion region.* All experimental cases were run using four models with different combinations of pre- and post-optimisation values of $\varGamma$ and $\xi$ (Table 4). The first model (baseline) provides the outputs of the simulation pre-optimisation: without an exclusion layer ($\xi =1$) and with the viscosity model parameters previously reported ($\varGamma =1$). This is compared to the fully optimised model, and models with optimised $\varGamma$ and pre-optimised $\xi$ and vice versa. The results of these simulations are used to investigate how much the inverse rheology process improved the results, what the relative contribution of $\varGamma$ and $\xi$ was and whether it differed between branches.

*Relative importance of shear rate and haematocrit in the viscosity model.* Two additional models were run for all cases: one with uniform haematocrit and one with uniform shear rate using the optimised $\varGamma$ and $\xi$ (see Table 5). For the uniform haematocrit model (green), the average value of the haematocrit was set throughout each branch, with 0 set in the exclusion layer. For the uniform shear model (red), a single value of shear rate, $\overline{{\dot{\gamma }}}=Q_p/w^3$ (often termed pseudo-shear rate), was entered into Eq. (18) throughout.

### Statistical analysis

**Fig. 5:**
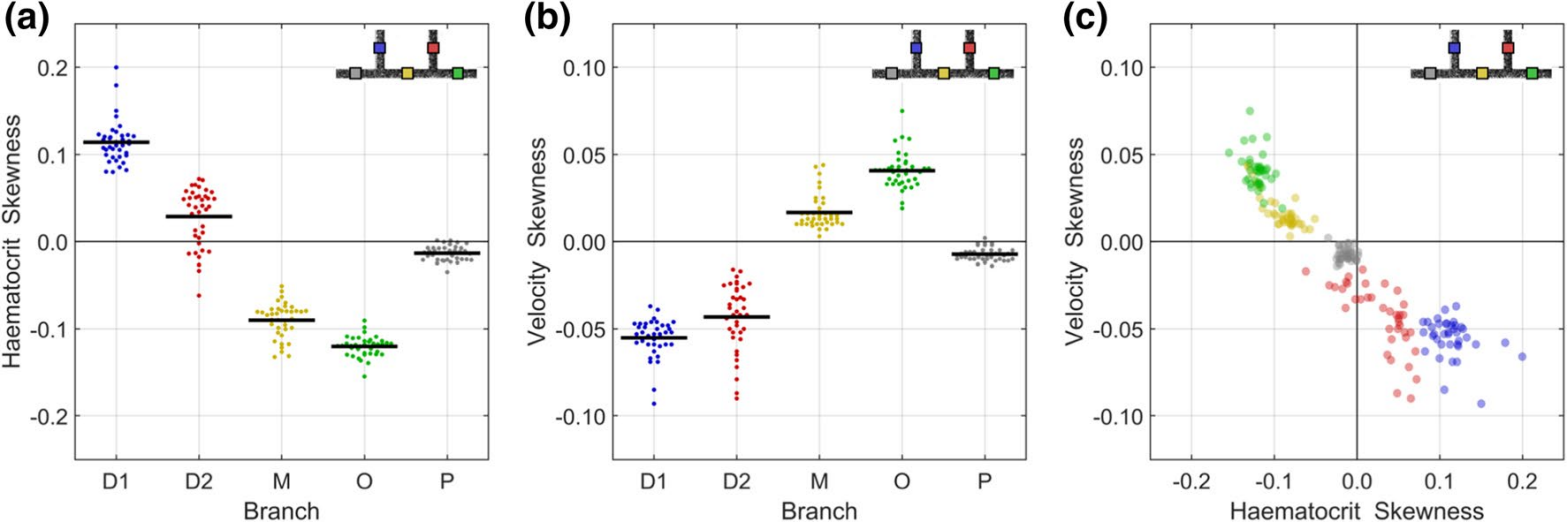
Characterisations of asymmetry of haematocrit (**a**) and velocity (**b**). **c** Demonstrates high degree of inverse correlation between the two parameters. Black lines show mean value. Branches are daughter 1 (D1), daughter 2 (D2), middle (M), outlet (O) and parent (P)

To analyse the difference between different models described in Tables 4 and 5, the nonparametric Kruskal–Wallis and Dunn–Sidak post hoc tests were applied. Pearson’s correlation coefficient was used for evaluating correlations between asymmetry indices (Fig. 5).

**Table 4:** Models for investigating the roles of $\varGamma$ and $\xi$

Model	$\varGamma$	$\xi \left( \upmu \mathrm{m}\right)$	Description
Baseline	1	0.3	Unmodified inputs
$\xi$-only	1	1.5	Exclusion layer modification only
$\varGamma$-only	3	0.3	Viscosity layer modification only
Optimised	3	1.5	Fully optimised model

**Table 5:** Models for evaluation of the relative importance of haematocrit and shear rate on the viscosity model

Model	Shear rate	Haematocrit	Description
$H,{\dot{\gamma }}$	Local	Local	Fully optimised model
$H,\overline{{\dot{\gamma }}}$	Uniform	Local	Uniform shear rate applied
${\overline{H}},{\dot{\gamma }}$	Local	Uniform	Uniform haematocrit applied

## Results

In order to provide a foundation for the inverse rheology process, Fig. 4 shows sample velocity, haematocrit and viscosity profiles. The outlet branch is selected for this sample case, in which the experimental velocity profile (shaded grey) is asymmetric, being skewed towards the outer wall (Fig. 4a). The cause of this asymmetry is the haematocrit distribution (Fig. 4c), which is skewed towards the inner wall, resulting in larger local viscosities for $n^*<0$ (Fig. 4f). Figure 4a shows the baseline model, corresponding to a haematocrit of zero immediately at the wall and the original viscosity model ($\xi =0.3\,\upmu \mathrm{m}$, $\varGamma =1$, purple). Although the model picks up some of the asymmetry and ‘bluntness’ of the experimental data, it is not blunt enough (as evidenced by the greater peak normalised velocity compared to the experimental data). The Newtonian velocity profile is provided for reference (black line) and is both symmetric and less blunt than the experimental data. As a point of comparison, the optimised model ($\xi =1.5\,\upmu \mathrm{m}$, $\varGamma =3$, blue) is provided (details of the optimisation results are provided Sect. 3.1). The optimised model recapitulates the experimental data far more effectively than the baseline model, although neither fully captures the velocity behaviour near the outer wall. To evaluate the performance of the modelling, Fig. 4d shows the error in the normalised velocity relative to the measured velocity at each vector location [Eq. (20)]. The optimised model is within the uncertainty of the experimental data for the majority of the velocity profile, whereas there are significant deviations for the baseline model.

At the other extreme, Figs. 4b and e show the equivalent plots for the maximal model, which demonstrates the upper bound of the amendments made in the optimisation process ($\xi =2.7\,\upmu \mathrm{m}$, $\varGamma =5$). The velocity profile is too blunt, with negative $e^*$ across most of the channel (Fig. 4e).

The viscosity profiles (Fig. 4f) provide insight into the cause of the differences in velocity between models. For all models, the viscosity profile is both asymmetric (due to haematocrit-dependent effects) and has a peak at $n^* \approx 0.05$, corresponding to the location of minimum shear rate at the apex of the asymmetric velocity profile. However, despite large differences in the magnitude of the predicted local viscosity in the channel centre, the apparent viscosity of all models is similar, with the baseline and maximal model having values 4% and 10% higher than the optimised model, respectively. This indicates that viscosity gradients play a larger role in determining the shape of the velocity profile than absolute values of viscosity.

As will become clear in the following analyses, the asymmetry in each branch of the channel plays a key role in determining the efficacy of the optimisation and the relative importance of the exclusion layer, viscosity model, haematocrit and shear rate. It is therefore useful to first consider the extent of asymmetry in each branch using the haematocrit and velocity skewness indices [Eqs. (12) and (13)] plotted in Fig. 5. For example, the haematocrit skewness in the outlet branch is negative (Fig. 5a, indicating a higher haematocrit for negative $n^*$, as shown for the sample case in Fig. 4c), with a mean value of $-0.12$. This value means that 62% of the RBCs are on the negative $n^*$ side of the axis, closer to the inner wall. The corresponding values of velocity skewness in the outlet branch are positive (Fig. 5b), with a mean value of 0.04, indicating that the average location of peak velocity is 0.04*w* away from the channel centre at $n^*=0.04$, consistent with Fig. 4a, b. There is negative correlation between the direction of haematocrit and velocity asymmetry (Fig. 5c) when analysing for all cases for the outlet branch, with a correlation coefficient of $-0.38$ ($p=0.02$). The same correlation is observed to varying degrees for all branches except the parent branch (which is symmetric, see Table 6). Considering all branches together, the correlation coefficient is $-0.94$ ($p<10^{-6}$), indicating that the asymmetry in the velocity distribution is predominantly dependent on the haematocrit distribution.

**Table 6:** Parameters for evaluation of relative importance of viscosity parameters

Branch	Correlation coefficient, $\rho$	*p*-value
All	− 0.94	$<10^{-6}$
Daughter 1	− 0.32	0.05
Daughter 2	− 0.72	$<10^{-6}$
Middle	− 0.81	$<10^{-6}$
Outlet	− 0.38	0.02
Parent	− 0.11	0.50

As the exclusion layer ($\xi$) is applied at all walls, it will inhibit asymmetry. By contrast, increasing $\varGamma$ increases local dependence on shear rate and hence enhances asymmetry. Both amendments will increase bluntness, by increasing the viscosity near the channel centre relative to the walls. Given the varying extents of asymmetry shown in Fig. 5, it is therefore expected that the optimal values of $\xi$ and $\varGamma$ may differ between branches.

### Inverse rheometry results

**Fig. 6:**
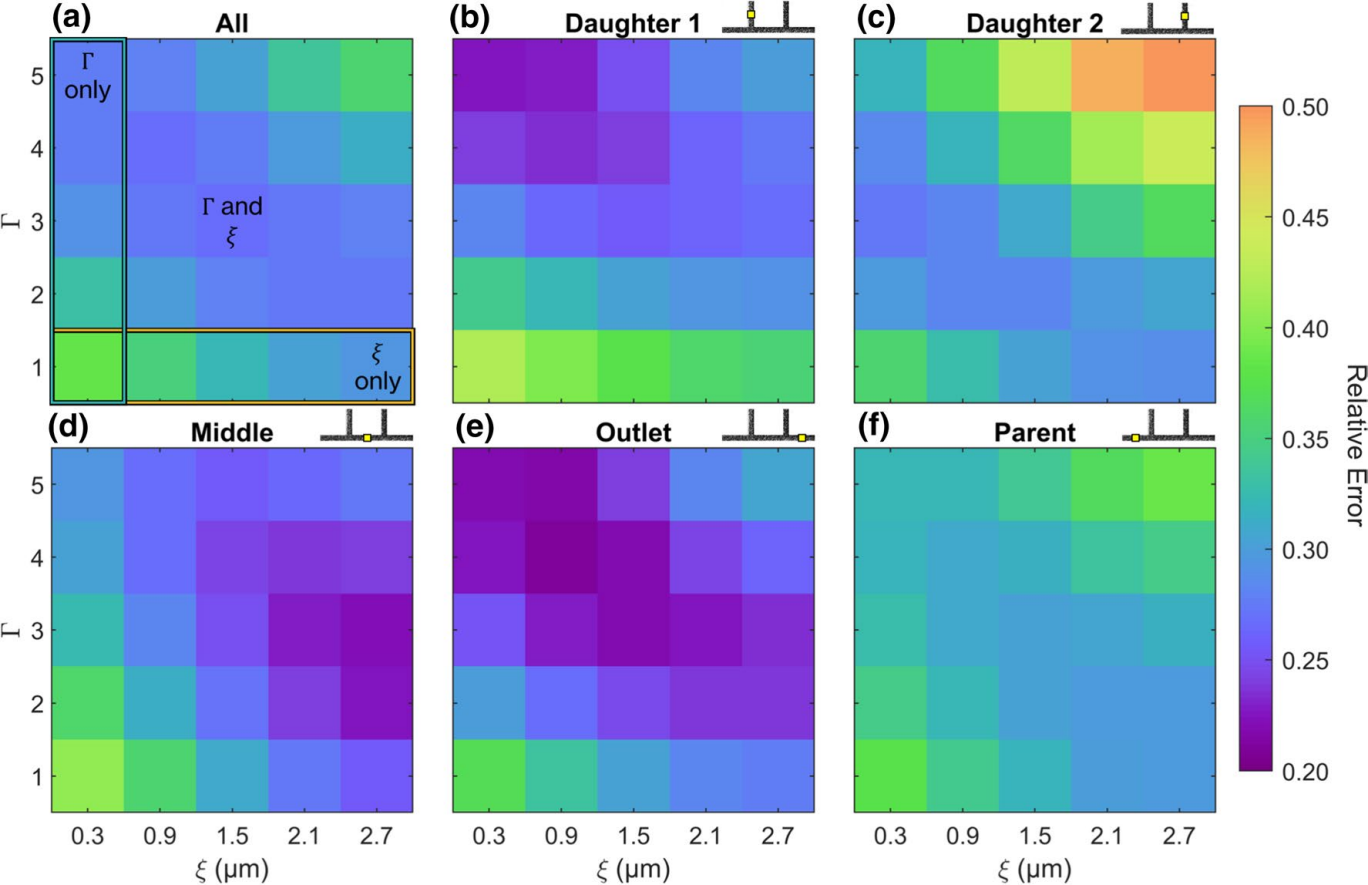
Outputs from inverse rheology approach, for all branches and each branch individually. $E^*$, is quantified as RMS of the error in normalised velocity (e.g. Fig. 4d, e) for the model relative to the Newtonian solution. Values shown are the average of all 8 sample cases. All display diagonal a trend, as expected because both modifications have the same effect on bluntness. Due to the characteristics of each branch, optimal parameters differ. **a** Average across all branches. $\varGamma =3$ and $\xi =1.5\,\upmu \mathrm{m}$ result in the minimum error, marked ‘$\varGamma$ and $\xi$’. Mustard and Teal boxes indicate what the tuning would have yielded if only $\xi$ or $\varGamma$ was considered, respectively. **b–f** Individual branches

Grids showing the average $E^*$, [Eq. (22)] over the 8 cases for the inverse analysis are shown in Fig. 6. As a result of the combined effects of $\xi$ and $\varGamma$ on bluntness, the minimum error values for each branch in Fig. 6 lie along the diagonal from top left to bottom right.

The flow in the parent branch follows a long ($\approx 200w$) straight section, so is symmetric, and thus, the experimental data are best captured using $\varGamma =1$ and $\xi =2.7\,\upmu \mathrm{m}$ (Fig. 6f). While it could be that the parent branch has a true cell-free layer, having had sufficient distance upstream for RBC migration to produce a CFL, this would not be the case in the other branches following perturbation of the RBC distribution in the bifurcations. Note that the absolute magnitude of $E^*$, in the parent branch is also higher than for other branches because the average error of the Newtonian profile (also being symmetric) is lower in this case.

Daughter Branch 1 is the most asymmetric branch (Fig. 5), and correspondingly high $\varGamma$ and low $\xi$ yield the best results (Fig. 6b). The daughter 2, middle and outlet branches all display characteristics somewhere between these two extremes (Fig. 6c–e). There is therefore not a single solution that is optimal for all branches, indicating a level of complexity not fully captured by the current modelling approach. To achieve a model that minimises error for all 5 branches on average, the mean across all branches was calculated (Fig. 6a). The average of all branches yields a minimum value of 0.267 at $\xi =1.5\,\upmu \mathrm{m}$, $\varGamma =3$, which is taken as the optimised model.

If the exclusion layer had not been accounted for (i.e. the first column of each panel in Fig. 6), then $\varGamma =5$ would have been selected (as it represents the smallest value in the first column of Fig. 6a). In this context, daughter 1 and outlet branches would perform well, while the parent, middle and daughter 2 branches would perform less well. Conversely, if the baseline viscosity model had been used (i.e. bottom row of each panel in Fig. 6), then $\xi =2.7\,\upmu \mathrm{m}$, would have been selected (as it represents the smallest value in the bottom row of Fig. 6a) and the daughter and outlet branches would perform much less well. In either case, the optimisation would not have converged, so would require more extreme modifications, which would amplify the differences between branches. Thus, the combination of both the exclusion layer and modification to viscosity model parameters reduces the error overall.

### Forward results

#### Evaluation of efficacy of inverse rheology

**Fig. 7:**
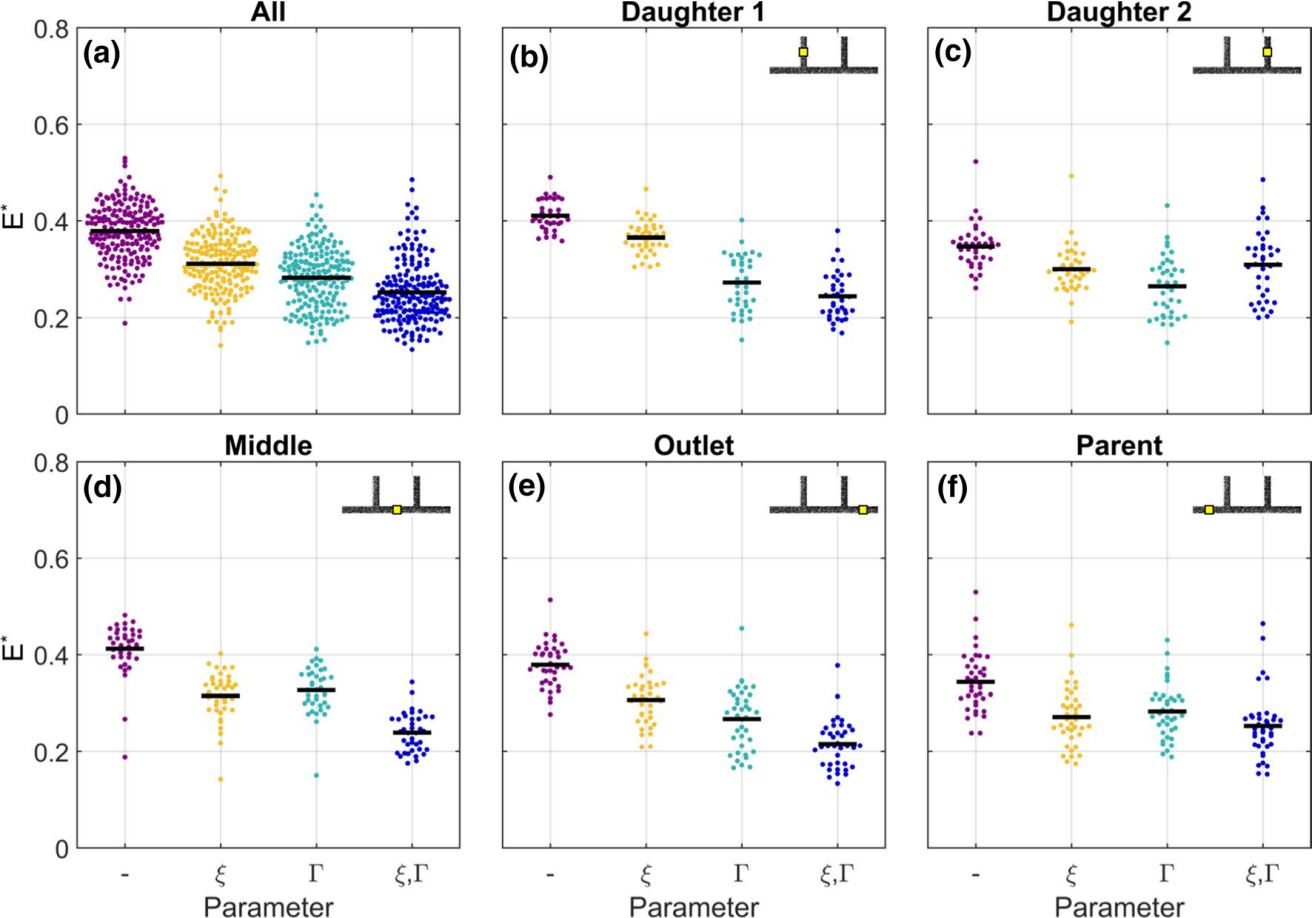
Beeswarm plots showing how the parameters $\xi$ or $\varGamma$ independently affect $E^*$, for all branches and each branch individually. Due to the characteristics of each branch, the relative importance of $\xi$ or $\varGamma$ differ. Horizontal black lines indicate mean

To further investigate how the parameters $\varGamma$ and $\xi$ interact in different branches, all 38 cases were simulated using the four models described in Table 4 (Fig. 7). The baseline model (purple) provides the results prior to optimisation. As demonstrated in Fig. 4a, these simulations reproduce some characteristics of the experimental data, but the predicted velocity profiles are not sufficiently blunt. The mean $E^*$, for the baseline model was 0.38, indicating a greater than 60% improvement relative to the Newtonian solution (Fig. 7a). The optimised model ($\xi ,\varGamma$: blue) had a mean $E^*$, of 0.25, with 0.31 and 0.28 for the $\xi$-only (yellow) and $\varGamma$-only (teal) models, respectively. The Kruskal–Wallis test with the Dunn–Sidak post hoc tests indicated statistically significant differences between all groups ($p<0.001$), indicating that both parameters contribute to the improvement in the performance of the modelling overall.

The relative importance of each parameter, however, varies between the branches. This can be interpreted from the reduction in $E^*$, for the $\xi$-only and $\varGamma$-only models (yellow and teal symbols, respectively) relative to the baseline model. For example, in daughter Branch 1 (Fig. 7b), the $\xi$-only model reduced $E^*$, only marginally and with borderline statistical significance ($p=0.03$). The $\varGamma$-only model, however, yields an average $E^*$ that is not significantly larger than for the optimised model ($p=0.7$).

For the middle and outlet branches (Fig. 7d, e), both single-parameter ($\xi$-only and $\varGamma$-only) models induce similar reductions in $E^*$ and are additive. (The optimised model performs better than either single-parameter model.) Differences between all models in these branches are statistically significant, except between the single-parameter models. In daughter Branch 2 (Fig. 7c), both the single-parameter models reduce the error ($p<0.001$), by a similar amount ($p=0.3$), but the combined model is less effective than either individual model and only marginally better than the baseline model ($p=0.02$). In the parent branch, the single-parameter models and the optimised model all significantly reduce $E^*$, compared to baseline, but they are not significantly different from one another ($p>0.1$).

In summary, the parent and daughter 2 branches (which have the lowest asymmetry) can be reasonably captured using either single-parameter model, and no improvement is achieved by the combination. However, for the three branches with the most asymmetry, optimisation of both $\xi$ and $\varGamma$ yields a reduction in $E^*$,. This highlights the importance of including data from branching networks, rather than straight channels only, when optimising fluid models for blood.

Despite the differences between the branches, it is clear from this analysis that the inverse rheology modelling (on a small subset of the data) is efficacious in better recapitulating the local haemodynamics than the baseline model, for which the exclusion zone is very small, and the viscosity model is based on previously defined parameters without modification.

#### Investigation of simulated haemodynamics in sequential bifurcations

**Fig. 8:**
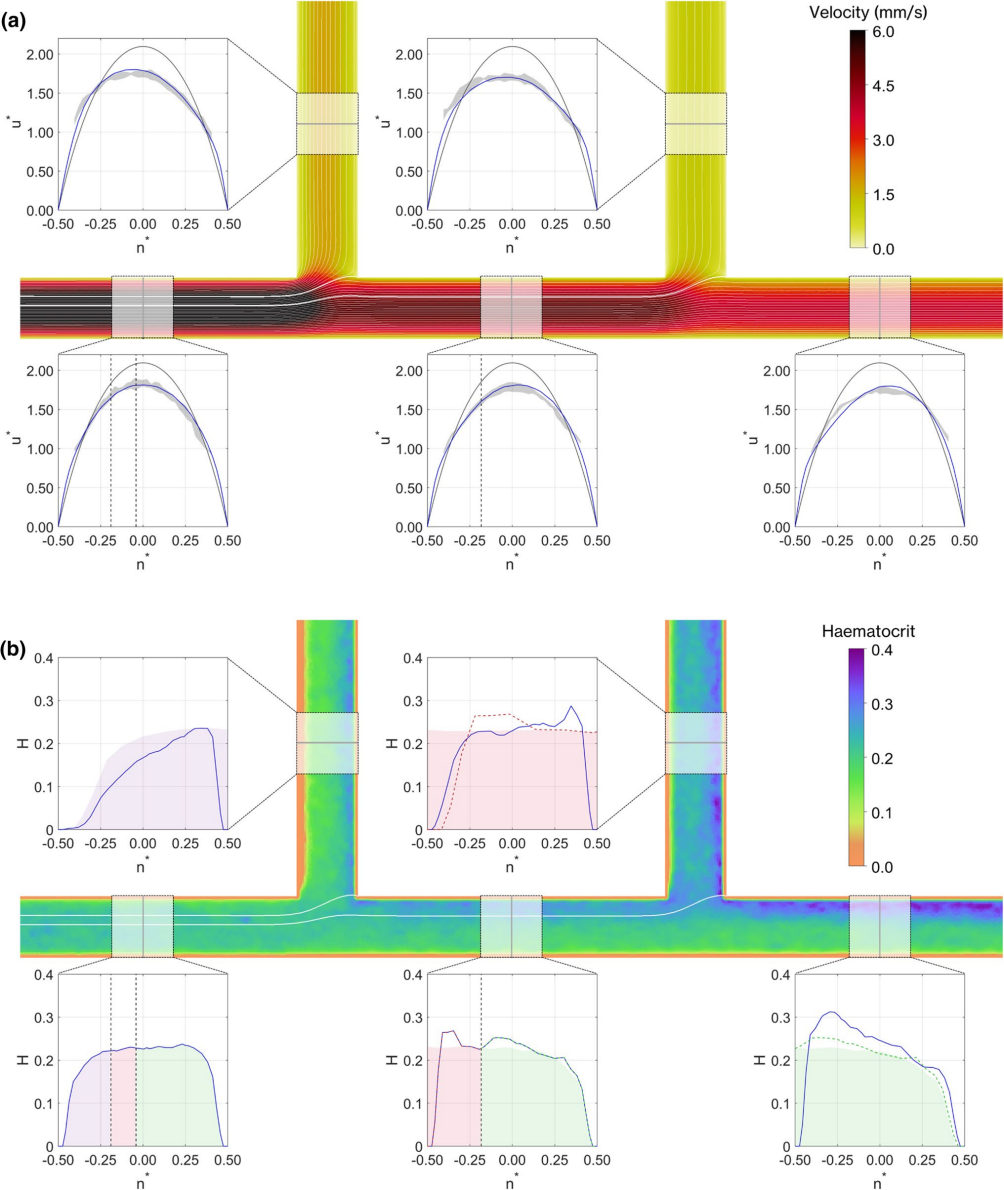
Velocity and haematocrit results, showing middle plane of the channel and profiles in each branch for sample case with $\overline{u_p}=3.8\,\mathrm{mm/s}$, $Q_1^*=0.25$ and $Q_2^*=0.25$ and the 9th lowest error averaged over all branches. **a** Normalised velocity profiles for final model (blue), Newtonian fluid (black)  and experiments (shaded grey). Dashed vertical black lines in parent branch indicate locations of separating streamlines. On the planar view, thin white lines indicate streamlines and the thick streamlines indicate the separating streamlines. **b** Haematocrit distribution in the centre plane. Blue lines show experimental measured distributions used in the model. Dashed vertical black lines in parent branch indicate separating streamlines. Coloured shading indicates mapping of haematocrit profile from parent branch (Bifurcation 1). Coloured dashed lines in middle, daughter 2 and outlet branches indicate mapping of haematocrit profile from middle branch (Bifurcation 2)

With the optimised settings, we consider the haemodynamics in the channel in a sample case with (${\overline{u}}_p =3.8\,\mathrm{mm/s}$, $Q_1^*=0.25$ and $Q_2^*=0.25$). The simulated velocity distributions for the whole domain are visualised in Fig. 8a, along with profiles in each branch. Comparing the simulated data (blue) with the experimental measurements (grey shading), the model performs well overall. The velocity in the parent branch is symmetric, but in all other branches, the velocity is skewed towards the outer wall of the bifurcation (negative $n^*$ for the daughter branches and positive $n^*$ for the middle and outlet branches). The white lines on Fig. 8a show the streamlines (calculated from the simulation), which aid in the visualisation of the flow distribution. The thick white lines (and vertical dashed black lines on the profiles) indicate the separating streamlines, which divide where the fluid separates into the daughter branches.

Figure 8b shows the haematocrit distribution throughout the domain, with the imposed $1.5\,\upmu \mathrm{m}$ exclusion layer visible as the thin orange band adjacent to all walls. At the apex of each of the bifurcations, the RBC concentration increases, and this region of elevated haematocrit near the inner wall persists downstream. In daughter Branch 1, the flow entering the branch is sampled from a symmetric distribution with a low haematocrit at the wall. Hence, in addition to the elevated haematocrit at the inner wall, there is a large region of low concentration at the outer wall. The shaded region indicates the approximate haematocrit distribution one would expect by advection alone based on simple conservation laws. This was produced by a simple mapping process based on the separating streamlines, given in the Supplemental Information with the sole purpose of providing an intuitive reference. The mapping predicts the downstream haematocrit distribution relatively well, but does not account for the region of reduced haematocrit at the inner wall ($n^*=0.5$) induced by the modelling of the exclusion layer. Similar characteristics can be observed in the middle branch.

In the branches of the second bifurcation (middle, daughter 2 and outlet), the dashed lines correspond to haematocrit mapping from the middle branch. In the outlet branch, the mapping from both the parent and middle branches are quite good matches of the measured profile (blue line), except the exclusion region at the inner wall. In daughter Branch 2, for which the flux from the parent branch was bounded on both sides by streamlines, the mapping from the parent branch (shaded region) predicts the average value, but not the exclusion layer at either wall. This analysis suggests that RBC advection alone predicts the distributions of RBC concentration moderately well for the present data, but it cannot reproduce the exclusion layers at the inner walls of the bifurcation.

**Fig. 9:**
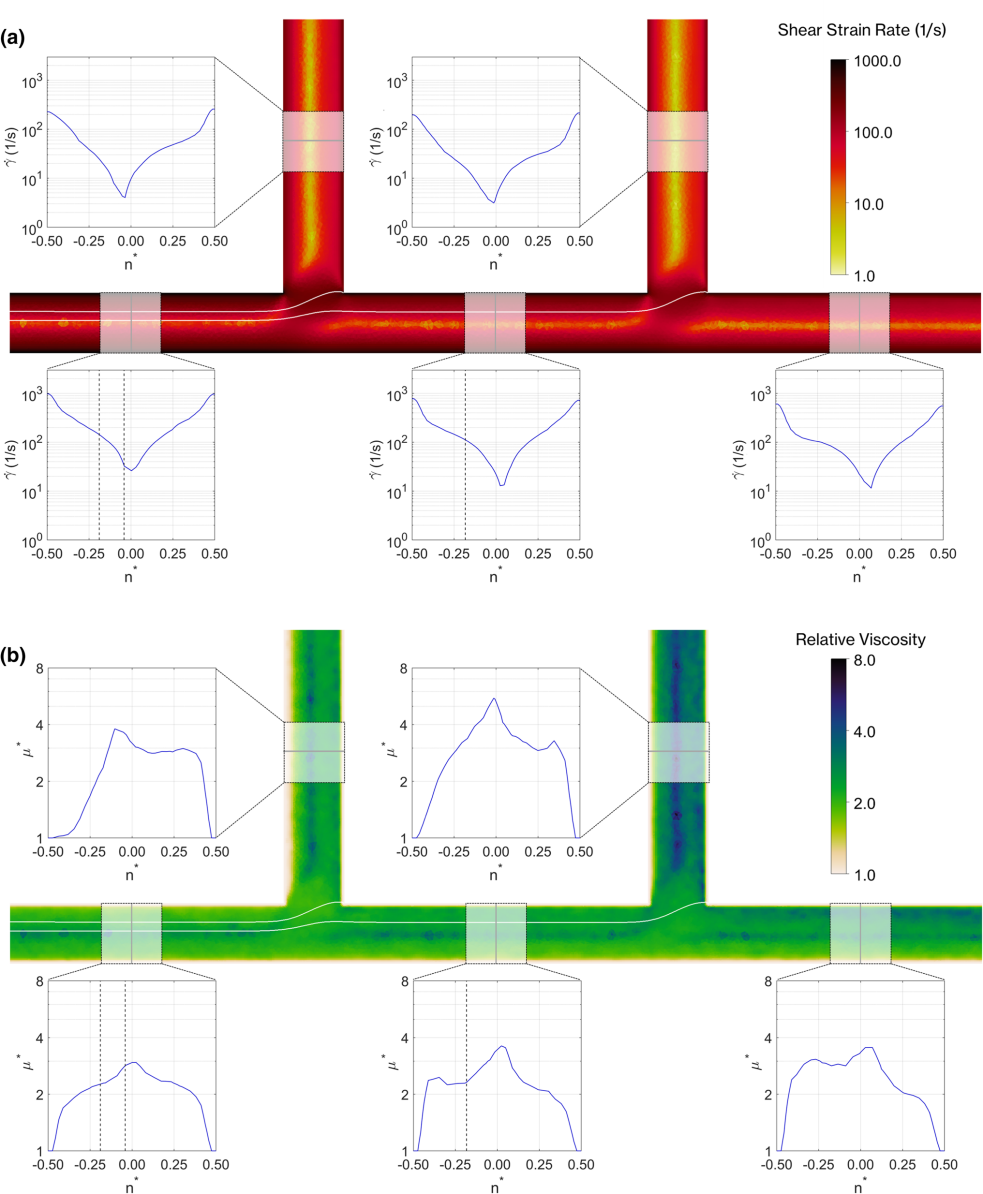
Shear rate magnitude and viscosity results, showing middle plane of the channel and profiles in each branch for samples case with $\overline{u_p}=3.8\,\mathrm{mm/s}$, $Q_1^*=0.25$ and $Q_2^*=0.25$. **a** Shear rate profiles for final model. Dashed lines in parent branch indicate locations of separating streamlines. On the planar view, thin white lines indicate streamlines and the thick streamlines indicate the separating streamlines. **b** Viscosity distribution in the centre plane. Dashed lines in parent branch indicate separating streamlines, and coloured shading indicates proportion of haematocrit profile that enters the downstream branches. Downstream branches show haematocrit from parent branch linearly mapped onto local coordinate system, as described in Supplemental Information: mapping analysis

The simulated distribution of shear rate at $z^*=0$ for the same case is shown in Fig. 9a. The shear rates vary in the range 1–1000 s$^{-1}$ and are asymmetric with minimum values in the range 3–30 s$^{-1}$ aligned with the location of the maximum velocity (Fig. 8a). Together, the haematocrit and shear rate distributions determine the distribution of viscosity predicted by the model (Fig. 9b). The viscosity profile in the parent branch increases gradually towards the channel centre, with a small peak corresponding to the location of minimal shear. In the other branches, there are two peaks: one corresponding to the location of minimum shear and the other corresponding to the location of maximum haematocrit. This analysis highlights the complexity of the haemodynamic environment and the challenge for continuum approaches: if the haematocrit is not accurately modelled, then estimating the viscosity distribution correctly will not be possible.

#### Importance of shear and haematocrit

**Fig. 10:**
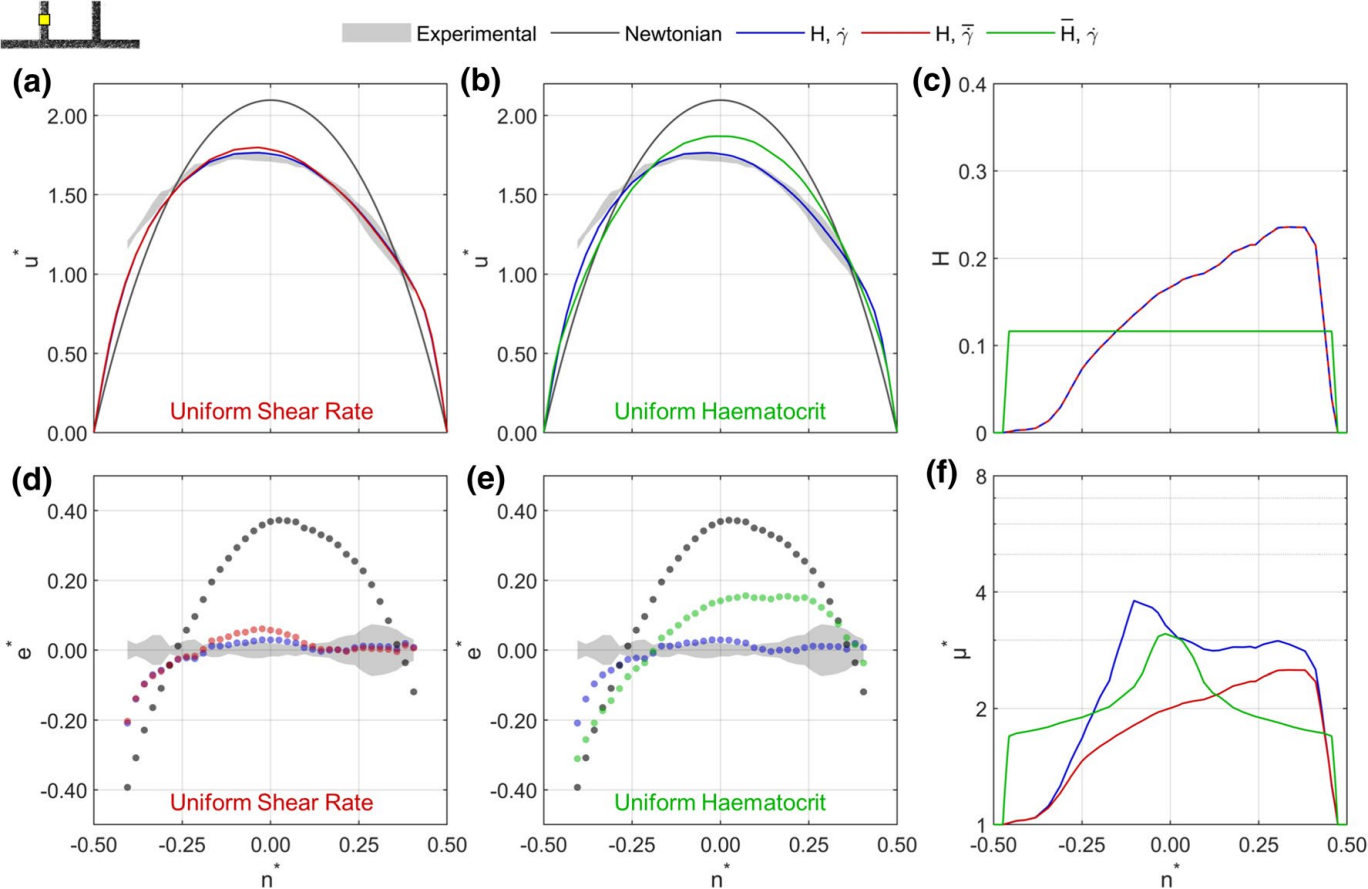
Sample normalised velocity profiles in daughter Branch 1 for a case with $\overline{u_p}=2.3\,\mathrm{mm/s}$, $Q_1^*=0.42$ and $Q_2^*=0.41$. Profiles using uniform shear rate (**a**) and uniform haematocrit (**b**) are compared to final parameters and the Newtonian solution. **d–e** Errors relative to the experimental data for the same cases. **c** Haematocrit profiles show how uniform haematocrit is applied, still including the exclusion zones. Uniform shear approach uses $\overline{{\dot{\gamma }}}=\overline{u_p}/w=w=46\,\mathrm{s}^{-1}$. **f** Viscosity profiles, showing the influence of each parameter in the predicted viscosity profile

Finally, we use the model to investigate the relative importance of haematocrit and shear rate in the modelling approach. Using the optimised model, simulations were carried out with uniform shear rate ($H,\overline{{\dot{\gamma }}}$) or uniform haematocrit (${\overline{H}},{\dot{\gamma }}$) (Table 5). The resulting velocity, haematocrit and viscosity profiles demonstrate the role of each parameter (Fig. 10). The model with uniform shear rate (red line) results in velocity profiles (Fig. 10a) that are very similar to the optimised model, with small deviations in the channel centre (Fig. 10d). The uniform haematocrit model, which still includes the exclusion layer (Fig. 10c), performs poorly, producing velocity profiles that are both symmetric and not sufficiently blunt (Fig. 10b, e). The differences between the viscosity profiles (Fig. 10f) clearly highlight the role of each of the two parameters.

**Fig. 11:**
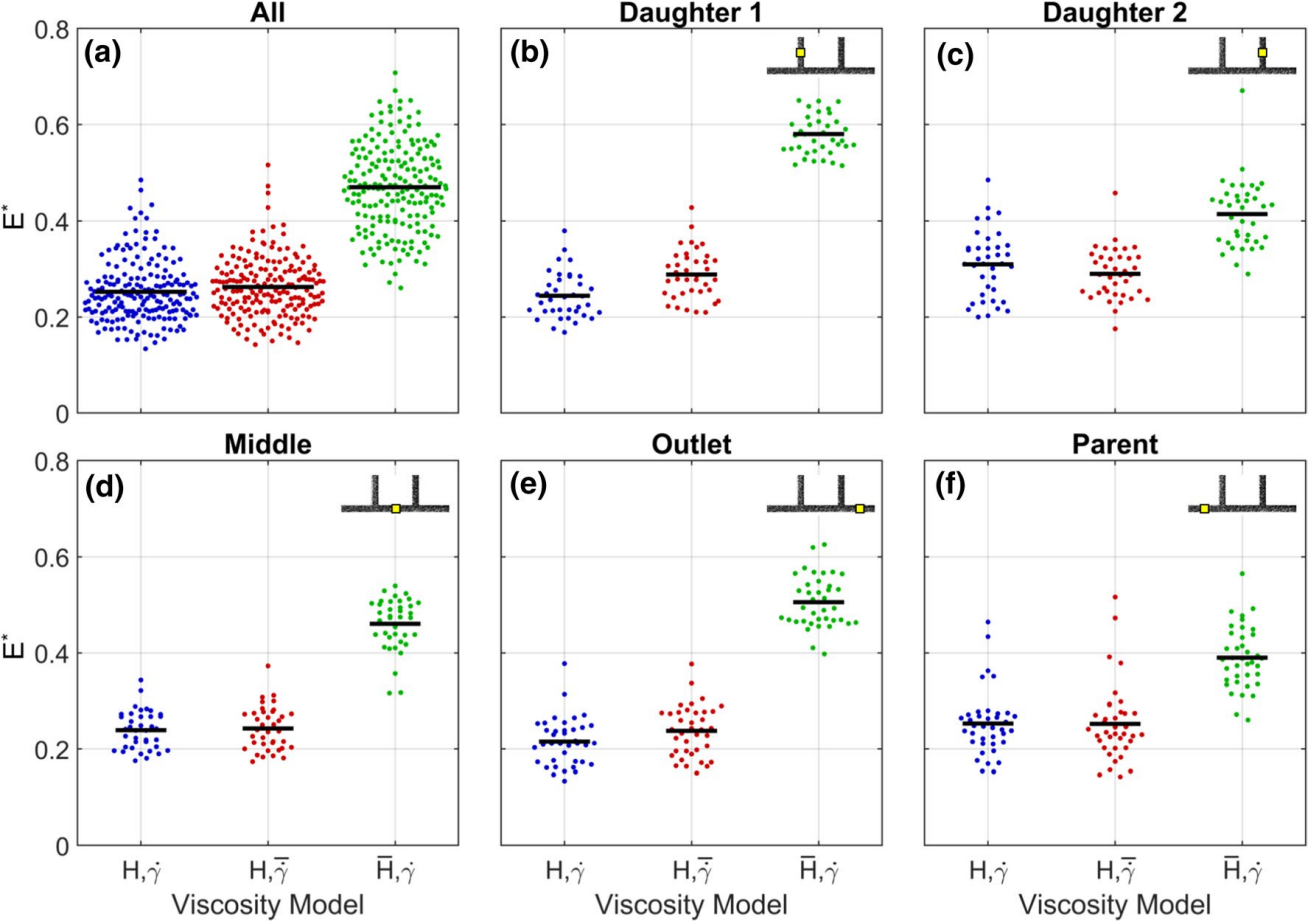
Beeswarm plots showing the relative importance of shear rate and haematocrit, for all branches and each branch individually. Due to the characteristics of each branch, the relative importance of shear rate and haematocrit differ. Horizontal black lines indicate mean

Considering all 38 cases, the uniform haematocrit model (${\overline{H}},{\dot{\gamma }}$) has a mean $E^*$ of 0.47 (Fig. 11), almost double the 0.25 for the optimised model ($p<10^{-6}$). The mean $E^*$, for uniform shear rate ($H,\overline{{{\dot{\gamma }}}}$) model was 0.26, which is not statistically different from the optimised model ($p=0.99$). This relationship holds in all branches. Furthermore, results from the optimised and uniform shear rate models are insignificantly different ($p>0.4$) for all branches except daughter Branch 1, which was marginally improved by accounting for shear rate distribution ($p=0.04$). Overall, this indicates that the spatial distribution of RBCs is likely the dominant factor in determining the viscosity distribution in microscale haemodynamics.

## Discussion

Blood rheology is extremely complex to model due to the complex interplay between RBC deformation, interactions of RBCs with each other and the vessel walls, and microstructures that form within the flow. Modelling approaches typically characterise blood viscosity as a single scalar, with a local value related to shear rate and volume fraction of the disperse phase (haematocrit). Due to the non-Newtonian nature of blood, models of its viscosity have typically been based on rheometry measurements in constant shear configurations (such as cone and plate or Couette rheometers), where a single shear rate can be explicitly stated for a given measured value of viscosity. However, due to the microstructural characteristics of blood and RBC migration in branching networks, such approaches may not be directly applicable. Although a number of groups have developed microstructural models (Giannokostas et al. 2021; Moyers-Gonzalez et al. 2008; Tsimouri et al. 2018), validation of these with experimental data has been limited to simple geometries.

Even with a validated viscosity model, a transport equation is also required to capture the heterogeneous distribution of RBCs. One of the most widely used models for multicomponent mixtures such as blood is the diffusive flux model developed by Leighton and Acrivos (1987) and further advanced by Phillips et al. (1992), which accounts for particle collisions and spatially varying viscosity. The model was originally developed for particles, but has been applied to blood flow in a number of studies (Chebbi 2018; Mansour et al. 2010; Schenkel and Halliday 2020). The model accounts for RBC flux with terms for diffusion induced by both viscosity gradient and shear rate, but there is no explicit term for particle migration away from the wall (although it can arise due to migration down a shear rate gradient). As formulated, the model therefore appears to be straightforward, with a single empirical parameter for each term along with an effective particle radius. However, Mansour et al. (2010) found that parameters that included distance from the wall were required, and it is likely that validation in complex geometries would generate need for further complications.

An alternative approach was implemented by Xu and Kleinstreuer (2019) who used a two-fluid model, with terms explicitly representing interaction between the phases, shear-induced diffusion and the lift force at the wall. Calibration of the empirical parameters in these models was carried out based on dedicated experiments to investigate lift (Abkarian et al. 2002; Grandchamp et al. 2013). This approach of determining terms for different RBC transport phenomena and tuning parameters based on prior studies is complicated by the fact that local haematocrit, shear rate and viscosity are intricately connected. Therefore, decomposition to obtain model parameters may produce numerical results that struggle to match experimental data in geometries with heterogeneous shear and haematocrit distributions.

To address the general problem of the interactions between RBC transport and viscosity, this study demonstrated a data assimilation approach for modelling blood flow in arteriolar-/venular-scale microvessels, which are too large for computationally expensive finite RBC modelling approaches and too complex for zero-dimensional network modelling approaches. We exploited high-resolution experimental data to reduce the parameter space and used an ‘inverse rheology’ approach in a geometry representing a small section of the microvasculature.

### Classification/terminology

We referred to this approach as ‘inverse rheology’, a term originally defined by Bandulasena et al. (2011) for a study with a simple shear thinning Xanthan gum solution. As blood rheology inherently involves the influence of RBC distribution, accounting for the exclusion layer within the optimisation approach under a definition of ‘rheology’ is appropriate. This method could be considered as a form of data assimilation, as it incorporates experimental and computational inputs to address shortfalls (Hayase 2015). An important distinction is that we do not combine the CFD and experimental data on a case-by-case basis, but rather use the average error between numerical and experimental data sets to choose optimal model parameters, which can then be applied to the main data set without further iteration. This approach is similar to the use of training data sets in machine learning approaches.

### Insights from the results

In the inverse phase, we optimised for two factors, the viscosity model and the size of the exclusion layer. The interaction between them required statistical analyses to choose an optimal model, which demonstrates the value of separating momentum and transport equations as proposed here.

#### Viscosity models

We used a single-phase continuum approach with the Quemada model to define the local viscosity, based on local haematocrit and shear rate. However, the general framework used in this study is equally applicable to other viscosity models that are fully empirical or incorporate microstructural components (Kaliviotis et al. 2018), viscoelasticity (Giannokostas et al. 2021) or other factors.

Comparison with data in long straight tubes (Chebbi 2018; Mansour et al. 2010; Moyers-Gonzalez et al. 2008; Sriram et al. 2014; Xu and Kleinstreuer 2019), a typical approach in model development, does provides some validation of model efficacy. However, as local viscosity and local haematocrit are inherently correlated, identifying a unique combination of transport and viscosity model parameters in straight vessels (wherein the viscosity and haematocrit are also spatially correlated) is impractical. A critical feature of the current study is the asymmetry in the haematocrit and velocity distributions, imparted by the bifurcating geometry, which is not captured in simpler models. Even so, the optimised value of $\varGamma$ in the current model should be interpreted as the value that minimises the numerical error for this specific blood sample in this particular geometry, rather than providing a ‘correct’ viscosity model for future experiments. Further investigation of different blood samples with viscosity directly measured and calculated using the optimisation process are required to improve understanding.

#### Exclusion layer

Our inverse approach also optimised for the size of the exclusion layer, which cannot be determined *a priori*, due to the deformability of RBCs and their different potential orientations near the wall (Fig. 3). The results yielded a parameter combination that minimised the average value across all branches, but demonstrated differences between branches depending on the extent of asymmetry. An ideal model would produce similar improvements for all branches, so it can be concluded that further development is required to improve upon the model applied in this study. One route to investigate will be whether the exclusion layer should be applied uniformly throughout the domain. In particular, at the inner walls of the bifurcation, RBCs are pushed against the wall and each other, and may thus infringe upon the exclusion layer thickness that was optimal for analysis overall. Better accounting for heterogeneity in this parameter could improve the performance of the model. A larger range of topographical layouts and improved near wall optical resolution would be necessary to support these developments.

It is worth restating that the exclusion layer used here is not the same thing as the cell-free layer (CFL) that is widely discussed. The origin of the CFL is migration of RBCs away from the vessel wall, which occurs over length scales of many channel widths (Carr and Xiao 1995; Pries et al. 1989; Ye et al. 2016). As such, immediately downstream of the bifurcation at the inner walls, there will not be a CFL, but there may be an exclusion zone. Given that RBCs can pack to concentrations of almost 100%, it is worth investigating whether the practical size of the exclusion zone is dependent on haematocrit and shear rate itself (due to their effects on RBC deformation).

#### Relative importance of shear and haematocrit

The results of the forward modelling (following optimisation) demonstrated that the most important parameter was the haematocrit distribution and that accounting for local distributions of shear rate did not significantly benefit the model in recapitulating the shape of the experimental velocity profiles. It may therefore be possible to use simpler viscosity models in which shear rate is a less critical parameter, which would simplify the analysis overall. Note that increasing $\varGamma$, while scaling the shear rate, can also be interpreted as increasing the relative importance of *H* for a given shear rate, explaining why optimisation of $\varGamma$ improved the model outcomes (Fig. 7). The magnitude of the shear rate is still an important parameter, as it determines both shear-induced migration and diffusion, and with pressure measurements to help validate viscosity calculations its specific role will become clearer.

#### Sensitivity of the Current Model

Regarding sensitivity of the optimisation, although the combination of $\varGamma$ and $\xi$ that produced the minimum average $E^*$, was selected, there were eight other combinations that were within 5%, and in particular $\varGamma =4$ and $\xi =0.9\,\upmu \mathrm{m}$ was within 2.5%. The selection of $\varGamma$ and $\xi$ could also be dependent on the method of error calculation, the specific cases selected for ‘training’ and the corresponding flow parameters, and statistical approaches used. Here, the mean was used rather than the median, as it is inherently more sensitive to outliers, so will reduce the maximum error from the cases used in the inverse stage. Overall, the selected optimal parameters could be sensitive to the specifics of the data set used, including number of branches, their relative asymmetry, etc. Machine learning tools, such as those recently demonstrated by Cai et al. (2021), may be an efficient route to a more robust optimisation.

### Limitations of the experimental data

Numerical models are only as good as their inputs, so it is important to consider uncertainties in the experimental data.

#### Particle image velocimetry for RBCs

Application of $\upmu$PIV algorithms to images of flowing RBCs is able to produce vector fields, but validation of the accuracy of the outcomes is difficult, due to the challenges of measuring local RBC velocities or even flow rates by other means. As the RBCs are not neutrally buoyant tracer particles and do not produce Gaussian distributions of illumination, the analyses used for true $\upmu$PIV data sets using tracer particles do not apply. Hence, parameters such as the depth of correlation (Olsen and Adrian 2000) are ill-defined. Poelma et al. (2012) highlighted how depth of focus depends on RBC *vs* tracer particles, magnification and velocity, but the results do not provide a framework that could easily be used to accurately re-scale measured velocities. Indeed, such a framework is impractical due to differences in optical properties between set-ups (light source and how it is focussed on the sample, *in vivo* *vs* *in vitro*, magnification, etc.). Here, we assumed that the calculated value of the velocity was 33% too low, corresponding to a depth of correlation that spans the whole depth of the channel (Poelma et al. 2012). This may mean that there is a systematic error in the absolute values of the velocities considered here. This is accounted for in comparisons between cases by normalising the velocity profiles. The possible error introduced by scaling the shear rate in the viscosity model is accounted for in the optimisation process by the factor $\varGamma$ which scales the shear rate in the model.

#### Velocity profile comparisons

Only a single velocity profile in each branch at a fixed distance from the bifurcation was used for comparing velocities between experimental and numerical models in the present study. This is because the whole channel was captured in a single frame, requiring a single *dt* for all branches despite the wide range of velocities present. By analysing each branch independently, an interrogation window with a long axial length enabled measurement of axial velocities over a large range, but such an approach would not work in the multidirectional flow around the bifurcations. For future studies, acquisitions with multiple *dt* values (Hain and Kähler 2007) would enable full domain measurements of sufficient resolution. Whole domain comparisons between numerical and experimental profiles could then provide more specific identification of optimal parameters.

#### Haematocrit measurement

The measurement of haematocrit distribution also has inherent uncertainties. Although a calibration process was carried out (Sherwood et al. 2014a), the input haematocrit was scaled based on red cell screening and Fåhraeus effect data from previous studies in different geometries (Gaehtgens et al. 1978; Pries et al. 1990). Counting of RBCs can be carried out at low concentrations ($<10$%) (Roman et al. 2016), but direct sampling of tube haematocrit would be required for higher levels. It is therefore possible that the actual tube haematocrit was higher or lower than reported here. Although this is not explicitly accounted for in the optimisation process, changing $\varGamma$ changes the relative importance of *H*, and thus implicitly accounts for it.

A major motivation to improve understanding and the ability to model microvascular blood flow is the investigation of how changes to RBC properties, such as increased aggregation and decreased deformability, play a role in blood flow regulation and dysregulation. The key mechanical stresses involved in this process are the transmural pressure and shear stress. *In vivo* measurement of either of these is currently impractical, so a numerical model, validated with experimental data, will provide valuable insight.

#### Square microchannel model

The experimental data used in the present study was acquired in square channels of constant width and depth, which is a simplification of the rounder cross sections and sequentially reducing diameters of *in vivo* microvessels. This limitation arises from the constraints of existing microfabrication processes to create round channels on the scale of microvessels. Micromilling with a ball-end bit (Carugo et al. 2012) can produce round channels down to $100\,\upmu \mathrm{m}$, but will become increasingly difficult for smaller sizes. Fenech et al. (2019) recently reported an elegant technique to produce channels of varying sizes with rounded cross section, but still with approximate right angles at the base. Fiddes et al. (2010) described a technique for introducing liquid silicone oligomer to a prefabricated microchannels to convert square cross-sectional straight channels into round ones. Abdelgawad et al. (2011) subsequently used a similar technique in more complex channels for cell trapping. This approach could potentially offer a solution for future studies.

The constant depth geometry used in the present study, however, proffers some significant advantages, in that it simplifies the haematocrit measurement (which would otherwise require correction for local channel depth) and allows the assumption of no $z^*$ axis forces. This is necessary for computing 3D distributions of haematocrit and velocity, and correspondingly flow rates. For round channels, this assumption would not be valid. Hence, an extension of the current experimental techniques to round channels would require PIV acquisition at multiple $z^*$ planes, as well as adjustments of the transmitted light data used to calculate the haematocrit for account channel depth. Work is currently underway to develop and validate such an experimental system.

Aside from the cross-sectional area, the microchannel used in this study had only straight channels without the tortuosity and curvature that will also affect the distribution of RBCs. Specific characterisation of the effects of these parameters on RBC velocity and haematocrit distributions will be carried out in future studies, along with investigations at length scales other than $50\,\upmu \mathrm{m}$.

#### Wall shear stress

Wall shear stress is a critical parameter in blood flow regulation, as it is sensed by endothelial cells as an input to balance vasodilatory (e.g. nitric oxide) and vasoconstrictive (e.g. endothelin-1) molecules that regulate vascular tone (Cho and Cho 2011; Schmetterer and Wolzt 1999). Wall shear stress cannot be measured, so must be inferred. It is the product of the local viscosity and shear rate, so is often calculated assuming plasma viscosity at the wall (Namgung et al. 2016; Reneman et al. 2006) or by assuming Poiseuille flow (Nagaoka and Yoshida 2006). However, as noted by Merrill (1969) the CFL is not always present. Although there may be an exclusion layer throughout, the velocity within it and the effect of RBCs pressed against or rolling along the wall is not clear. Furthermore, the endothelial surface layer will also play a key role *in vivo* (Potter and Damiano 2008; Pries 2005) and so even an accurate model of the blood itself will not provide the full picture. Nonetheless, experimental measurements of pressure drop (which can provide information via a force balance with WSS) will provide further insight into true values of WSS. The current study is also limited to producing time- or phase-averaged values of WSS and cannot reproduce the transient fluctuations introduced by individual RBCs, which can be significantly higher than time-averaged values (Freund and Vermot 2014). However, whether such transient WSS fluctuations on endothelial cells have the physiological importance requires further investigation.

#### Pressure distribution

The pressure distribution is important because the transmural pressure (the pressure across the vessel wall) is the main input signal for the myogenic response, whereby arteriolar smooth muscle cells contract in response to increased pressure to help regulate vascular tone. Pressure distributions in the present model were predicted within the model, but not analysed further, because experimental data on these is required for further validation. The apparent viscosity is inherently related to the pressure distribution and correspondingly to the branch-wise distribution in average haematocrit (i.e. the Zweifach–Fung ‘law’). Knowledge of the pressure will therefore become more important in larger networks and when modelling RBC transport.

#### Different blood samples

Changes to the behaviour of RBCs have been reported for various microvascular diseases Agrawal et al. (2016); Connes et al. (2016); Depond et al. (2020); Dintenfass (1982). For example, in diabetes, RBC deformability is decreased due to glycosylation of the RBC membrane and cytoplasm (Tomaiuolo 2014; Vekasi et al. 2001), and RBC aggregation is increased (Cho and Cho 2011; Le Dévéhat et al. 2004; Kostova et al. 2012), likely due to elevated plasma fibrinogen levels (Bembde 2012).

Whether a simple model such as that applied here is able to capture changes in deformability and aggregation remains to be seen. Chien (1970) proposed that the two phenomena both affect blood viscosity via the same mechanism: a change in the effective cell volume. The current approach provides a framework to investigate this idea and thus whether the haematocrit distribution alone is sufficient or whether additional model complexity is required.

#### Transport modelling within the Inverse Framework

The approach demonstrated here is of course, only half of the story, being dependent on experimental measurements of haematocrit distribution as an input for the simulations. The next step is development of a similar process to optimise empirical parameters for continuum RBC transport. Such models must account for RBC migration away from vessel walls, the effects of collisions with other RBCs and the effects of local viscosity as described above. Each of these model components requires empirical parameters, which also need to be optimised. Optimising at the same time as the viscosity parameters would be difficult due to the interdependence between the parameters (Schenkel and Halliday 2020) and the sheer size of the parameter space. Rather, first optimising rheometric parameters based on the measured haematocrit distributions (as in this study), then using the optimised viscosity model in an optimisation of transport parameters that match experimentally measured haematocrit distributions should yield clear outcomes. This two-step process would provide a full continuum model that could be optimised on sample geometries and then applied to different network geometries without the need for further experimental inputs. For example, the model could be tuned for a given blood sample using *in vitro* geometries, then applied to 3D networks created from segmentation of *in vivo* imaging data. This has the potential to provide a method for efficient and personalised modelling of haemodynamics in complete microvascular networks.

### Conclusions

Modelling microscale blood flow is a challenging task due to the complexity of both the blood as a fluid and the branching networks in which it flows. A large number of numerical methods have been proposed with varying degrees of complexity and dependence on empirical parameters, but have been inherently limited by the availability of high-quality experimental data in geometries other than long straight tubes. In this study we optimised a simple continuum mixture model, using an inverse rheology approach that incorporates experimental data in the process from the beginning. Comparison with a larger data set allowed us to quantitatively evaluate how well the model works in a complex geometry. Although there is much to do to develop the model into a practical tool, the approach described in this study will provide a framework for this development and makes a step towards better incorporation of experimental and computational data in the study of microhaemodynamics.

## Supplementary Information

Below is the link to the electronic supplementary material.

Supplementary material 1 (pdf 1163 KB)
