# Supplementary material for: Continuum microhaemodynamics modelling using inverse rheology
Source: Biomech Model Mechanobiol. 2021 Dec 14;21(1):335–61. doi: 10.1007/s10237-021-01537-2 (PMC8807439; doi:10.1007/s10237-021-01537-2)
Supplement: Supplementary file 1 — Supplementary material 1 (pdf 1163 KB) [file 10237_2021_1537_MOESM1_ESM.pdf]

Supplementary Information

Continuum microhaemodynamics modelling using inverse rheology

Joseph van Batenburg-Sherwood and Stavroula Balabani

Conservation of Mass and RBC flux

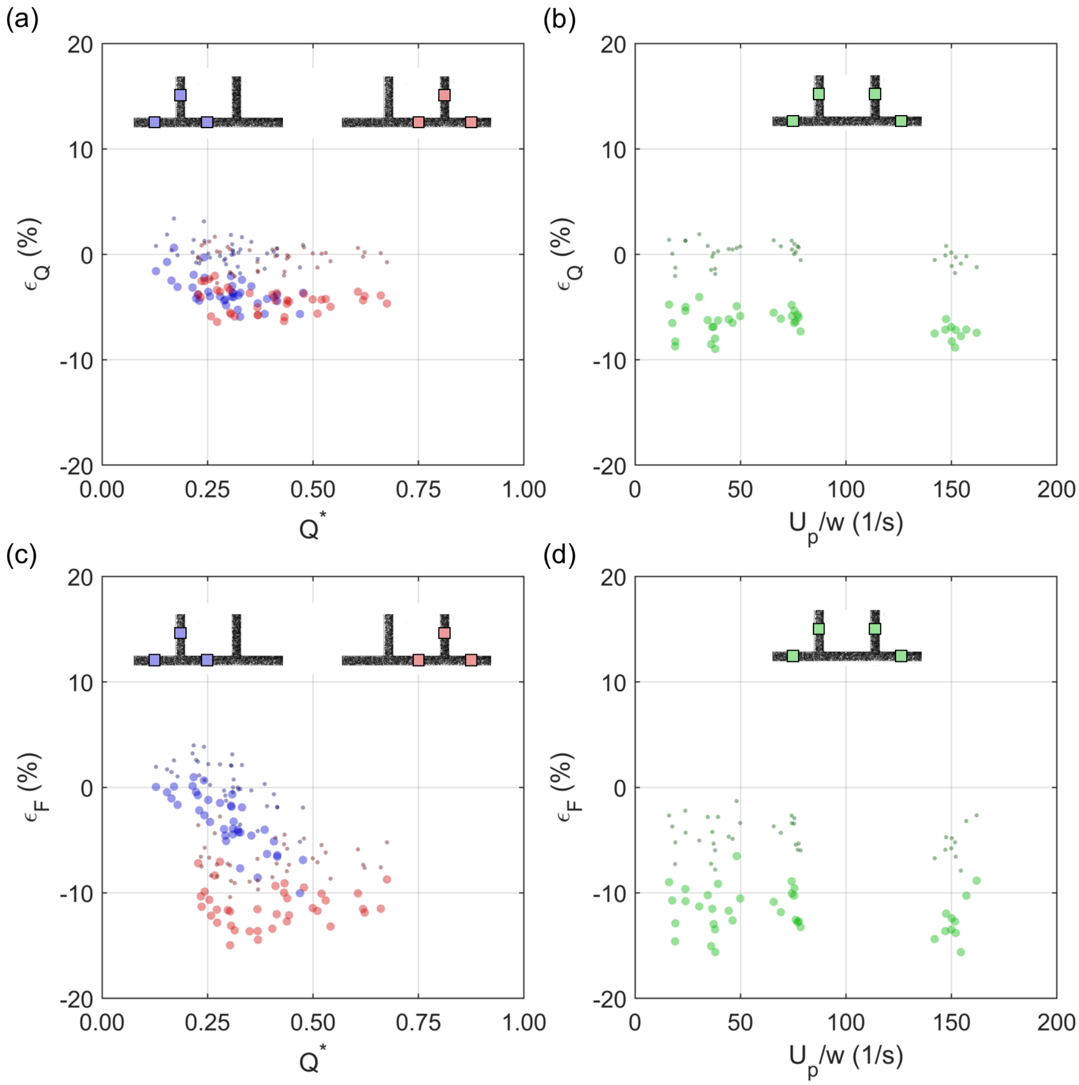

Figure S1: Conservation errors in experimental data. a) Conservation of mass error for separate bifurcations against flow ratio for Bifurcation 1 ($e_{Q,1}$, blue) and Bifurcation 2 ($e_{Q,2}$, red). b) Conservation of mass error for the whole domain, $e_{Q,tot}$, against parent branch velocity in channel widths/second. c) and d) show equivalent plots for error in conservation of RBC flux. Large circles indicate raw data and small dots show data following empirical 'correction' of raising velocities to the power 1.066.

Viscosity Model Parameters

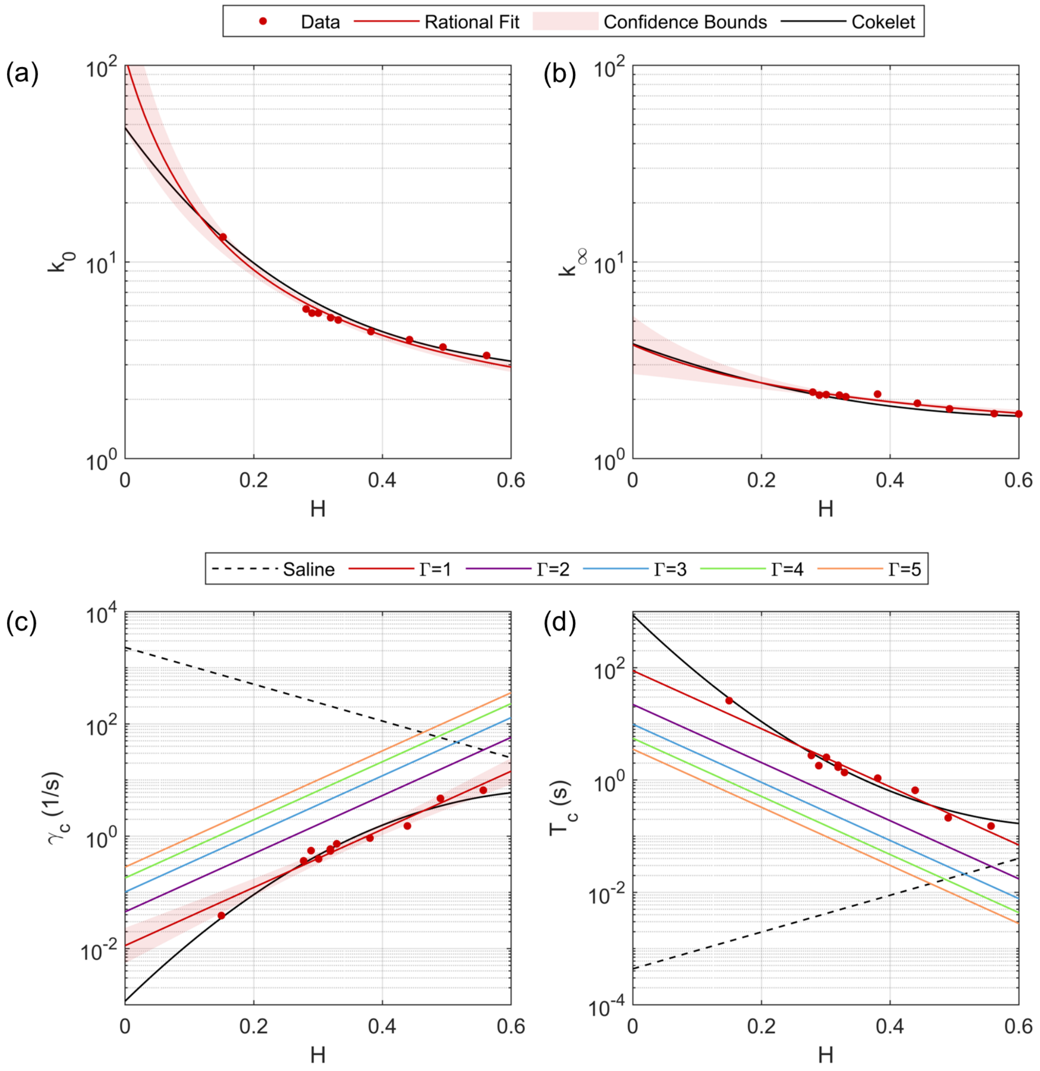

Figure S2: Parameters used in the empirical viscosity model. Rational fit (Table 2) compared to original fits from Cokelet (1987) for intrinsic viscosity at a) low shear, b) high shear. c) Comparison of linear and quadratic (Cokelet, 1987) fits for critical shear rate, and how this parameter is modified by Γ. d) Timescale of shear dependent behaviour (1/critical shear) and how it is affected by Γ.

Mesh Sensitivity

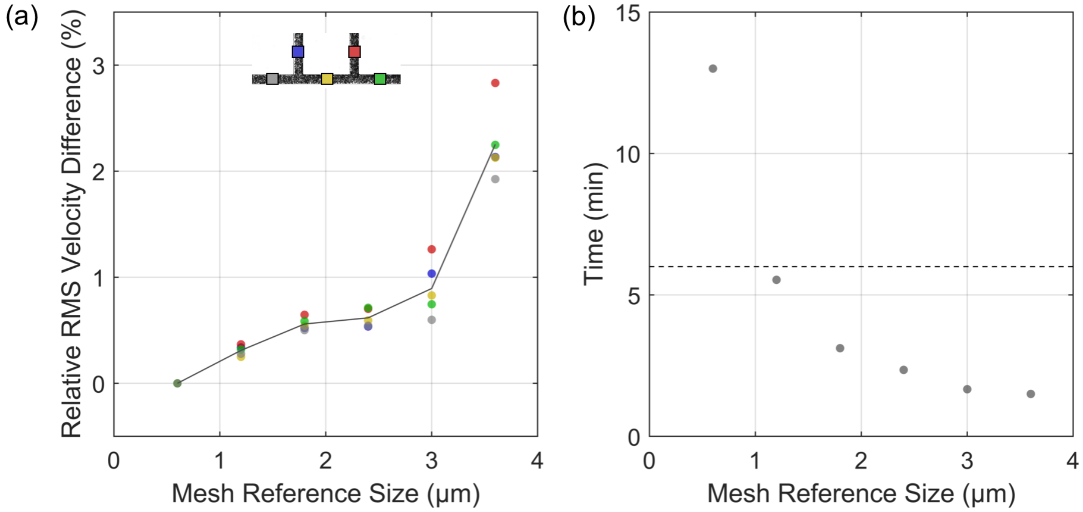

Figure S3: Mesh sensitivity analysis. The sample case shown in Figures 7 and 8 ($\overline{u}_p = 3.8mm/s$, $Q_1^* = 0.25$ and $Q_2^* = 0.25$) was evaluated with multiple meshes, as described in Table S1. a) The RMS of the difference between the finest mesh and all other meshes, used to indicate sensitivity to further refinement. b) Simulation time for each of the mesh resolutions. Dashed line indicates time to load the experimental data (equal for all cases).

Table S1: Details of the mesh inputs and outputs from the mesh sensitivity analysis. Bold indicates final mesh selected.

	Reference size (μm)	First inflation layer height (μm)	Number of elements	Simulation time (minute:sec)	Relative RMS velocity difference (%)
Finest	0.6	0.3	6,213,348	13:00	-
**Finer**	**1.2**	**0.6**	**2,361,885**	**05:32**	**0.31**
Fine	1.8	0.9	954,115	03:07	0.56
Medium	2.4	1.2	563,505	02:21	0.62
Coarse	3.6	1.8	214,383	01:40	0.89
Coarsest	4.8	2.4	139,722	01:30	2.25

Mapping Analysis

For Figure 8b, a 'mapping' process is applied to demonstrate how RBC concentration from the upstream branches would be distributed in the downstream branches, assuming that the RBCs do not disperse across streamlines and behave as non-interacting particles that do not influence local density. As RBCs are $\approx 10\%$ denser than plasma, for haematocrit up to 30% the density would vary by 3% at most across the domain, so the latter assumption is appropriate.

Considering a control volume bounded by two streamlines in the centre plane, and applying Reynolds transport theorem for i) mass and ii) number of RBCs yields

$$\int u_U \, dA_U = \int u_D \, dA_D \tag{S1}$$

$$\int u_U H_U \, dA_U = \int u_D H_D \, dA_D \tag{S2}$$

where the subscripts U and D indicate the upstream and downstream limits of the control volume, u is velocity normal to the control surface and H is haematocrit. Assuming an infinitesimal distance between streamlines such that u and H do not change across A, Equations S1 and S2 reduce to

$$u_U A_U = u_D A_D \tag{S3}$$

$$u_U H_U A_U = u_D H_D A_D \tag{S4}$$

Under these assumptions, $H_U = H_D$, irrespective of A_U and A_D (the latter will always be larger in the current geometry with equal branch sizes). The haematocrit is therefore constant along a streamline under these assumptions.

Consider a control volume in the Parent Branch bounded by two separating streamlines at $n^*_{p,1}$ and $n^*_{p,2}$ that feeds Daughter Branch 1. The downstream end of the control volume will occupy the entire branch $-0.5 < n^*_D < 0.5$. Assuming distances between streamlines do not change significantly, a mapped transverse co-ordinate n^*_{map} can be calculated according to:

$$n^*_{map} = \frac{n^*_U - n^*_{p,1}}{n^*_{p,2} - n^*_{p,1}} - 0.5 \tag{S5}$$

and the distribution of haematocrit $H_U\left(n^*_U\right)$ can then be plotted against n^*_{map} to generate an estimate of the haematocrit distribution in the downstream branch. A similar mapping can be applied to other branches of the bifurcation.
